# Vitamin B_1_ diversity and characterization of biosynthesis genes in cassava

**DOI:** 10.1093/jxb/erx196

**Published:** 2017-06-21

**Authors:** Nathalie Mangel, Jared B Fudge, Teresa B Fitzpatrick, Wilhelm Gruissem, Hervé Vanderschuren

**Affiliations:** 1 Department of Biology, ETH Zurich, Zurich, Switzerland; 2 Department of Botany and Plant Biology, University of Geneva, Geneva, Switzerland; 3 AgroBioChem Department, Gembloux Agro-Bio Tech, University of Liège, Gembloux, Belgium

**Keywords:** Cassava, crop, diversity, vitamers, vitamin B_1_, riboswitch

## Abstract

Vitamin B_1_, which consists of the vitamers thiamin and its phosphorylated derivatives, is an essential micronutrient for all living organisms because it is required as a metabolic cofactor in several enzymatic reactions. Genetic diversity of vitamin B_1_ biosynthesis and accumulation has not been investigated in major crop species other than rice and potato. We analyzed cassava germplasm for accumulation of B_1_ vitamers. Vitamin B_1_ content in leaves and roots of 41 cassava accessions showed significant variation between accessions. HPLC analyses of B_1_ vitamers revealed distinct profiles in cassava leaves and storage roots, with nearly equal relative levels of thiamin pyrophosphate and thiamin monophosphate in leaves, but mostly thiamin pyrophosphate in storage roots. Unusually, the cassava genome has two genes encoding the 4-amino-2-methyl-5-hydroxymethylpyrimidine phosphate synthase, THIC (*MeTHIC1* and *MeTHIC2*), both of which carry a riboswitch in the 3ʹ-UTR, as well as the adenylated thiazole synthase, THI1 (*MeTHI1a* and *MeTHI1b*). The *THIC* and *THI1* genes are expressed at very low levels in storage roots compared with the accumulation of vitamin B_1_, indicating only limited biosynthesis *de novo* therein. In leaves, vitamin B_1_ content is negatively correlated with *THIC* and *THI1* expression levels, suggesting post-transcriptional regulation of *THIC* by the riboswitch present in the 3ʹ-UTR of the *THIC* mRNA and regulation of *THI1* by promoter activity or alternative post-transcriptional mechanisms.

## Teaser

Vitamin B_1_ biosynthesis *de novo* is regulated by duplicated genes and a conserved riboswitch in cassava. Diversity of vitamin B_1_ levels in cassava leaves negatively correlates with expression of selected vitamin B_1_ biosynthesis genes.

## Introduction

Vitamin B_1_ is essential for all living organisms. It functions as a cofactor for various enzymes involved in key metabolic pathways, including glycolysis, the citric acid cycle, branched-chain amino acid biosynthesis, and the cytosolic non-oxidative stage of the pentose phosphate pathway (Goyer, 2010; Rapala-Kozik, 2011). Mammals, including humans, lack the ability to biosynthesize vitamin B_1_ and therefore crop plants are one of the major dietary sources of this micronutrient. In humans, an acute lack of vitamin B_1_ can lead to various chronic diseases, including cardiovascular diseases known as ‘wet’ beriberi and neurological disorders termed ‘dry’ beriberi (Rapala-Kozik, 2011). Globally, nearly two billion people suffer from deficiencies in one or more essential micronutrients (Thompson and Amoroso, 2011; von Grebmer *et al.*, 2014; Singh *et al.*, 2016); this is especially the case in low- and middle-income countries whose populations have low dietary diversity and limited access to supplementation strategies (Muthayya *et al.*, 2013). Although rice, maize, and wheat remain the world’s leading cultivated crops, cassava is the most widely grown orphan food crop and is consumed predominantly in developing countries (Sayre *et al.*, 2011; Varshney *et al.*, 2012). Raw cassava storage roots have a vitamin B_1_ level that only partially covers the human daily requirement (Fitzpatrick *et al.*, 2012). Moreover, cassava leaves and storage roots are usually soaked and boiled in water for the purpose of cyanide detoxification before consumption (Padmaja, 1995; Ufuan Achidi *et al.*, 2005; Muoki and Maziya-Dixon, 2010). This processing can affect the nutritive value through modification and losses of nutrients, including the water-soluble and/or heat-labile vitamins (Montagnac *et al.*, 2009*b*; Li *et al.*, 2015).

Vitamin B_1_ biosynthesis *de novo* in plants has been mostly characterized in the model plant Arabidopsis (Goyer, 2010; Rapala-Kozik, 2011; Fitzpatrick and Thore, 2014). Vitamin B_1_ is present as three predominant vitamers in the cell, namely thiamin, thiamin monophosphate (TMP), and thiamin pyrophosphate (TPP). Triphosphorylated and adenylated forms of thiamin also exist in plants and animals but are far less abundant (Bettendorff *et al.*, 2007; Gangolf *et al.*, 2010). TMP is generated by the fusion of 4-methyl-5-β-hydroxyethylthiazole phosphate (HET-P) and 4-amino-2-methyl-5-hydroxymethylpyrimidine pyrophosphate (HMP-PP), which are independently biosynthesized. Biosynthesis of the thiazole moiety in plants is assumed to occur via a pathway similar to that described in yeast, in which adenosine diphospho-5-(β-ethyl)-4-methylthiazole-2-carboxylic acid (ADT) synthase (THI4) catalyzes the conversion of NAD^+^, glycine, and a sulfur atom from the THI4 protein itself to the adenylated thiazole intermediate, ADT (Chatterjee *et al.*, 2011; Goyer, 2017). ADT is subsequently transformed to HET-P by an uncharacterized NUDIX hydrolase (Goyer *et al.*, 2013; Goyer, 2017). ADT synthase is a single-turnover enzyme, which after the donation of sulfur is assumed to become catalytically inactive with regard to thiamin biosynthesis (Chatterjee *et al.*, 2011; Fitzpatrick and Thore, 2014). Based on sequence homology, orthologs of *THI4* (named *THI1*) have been characterized in several plant species (Belanger *et al.*, 1995; Machado *et al.*, 1996; Wang *et al.*, 2006). The vitamin B_1_ pyrimidine moiety in plants is biosynthesized via a pathway similar to the one characterized in bacteria, during which 4-amino-2-methyl-5-hydroxymethylpyrimidine phosphate (HMP-P) synthase (THIC) catalyzes a complex rearrangement of 5-aminoimidazole ribonucleotide (AIR) to HMP-P (Lawhorn *et al.*, 2004; Raschke *et al.*, 2007; Kong *et al.*, 2008; Coquille *et al.*, 2013). In plants, HMP-P is further phosphorylated to HMP-PP by a bifunctional enzyme named TH1 in Arabidopsis (Ajjawi *et al.*, 2007*b*) and THI3 in maize (Rapala-Kozik *et al.*, 2007), which subsequently catalyzes the condensation of HMP-PP and HET-P to TMP. In plants, biosynthesized TMP is first dephosphorylated to thiamin and subsequently pyrophosphorylated to TPP. Plant enzymes from the haloacid dehalogenase (HAD) phosphatase family have recently been demonstrated to have a TMP-selective phosphatase activity, with the enzyme TH2 purportedly specific for TMP (Hasnain *et al.*, 2016; Mimura *et al.*, 2016). The conversion of thiamin to TPP is catalyzed by thiamin pyrophosphokinase (TPK) (Rapala-Kozik *et al.*, 2009). Most of the enzymes involved in vitamin B_1_ biosynthesis *de novo* are localized in the chloroplast (Belanger *et al.*, 1995; Chabregas *et al.*, 2001; Ajjawi *et al.*, 2007*b*; Raschke *et al.*, 2007; Kong *et al.*, 2008), except for TPKs, which are localized in the cytosol (Ajjawi *et al.*, 2007*a*), and TH2, which is also cytosolic as well as being potentially targeted to the mitochondria (Mimura *et al.*, 2016).

In Arabidopsis, *THIC* transcript levels are regulated by light (Raschke *et al.*, 2007), the circadian clock (Bocobza *et al.*, 2013), and a riboswitch in the 3ʹ-UTR of *THIC* mRNA (Sudarsan *et al.*, 2003; Bocobza *et al.*, 2007, 2013; Wachter *et al.*, 2007), which collectively contribute to the regulation of vitamin B_1_ biosynthesis. The current model states that the *THIC* riboswitch undergoes alternative splicing in the 3ʹ-UTR region as a function of TPP, leading to the formation of transcripts with different 3ʹ-UTR lengths that affect mRNA stability (Bocobza *et al.*, 2007; Wachter *et al.*, 2007). When the intracellular TPP concentration is high, binding of this ligand to the riboswitch changes its conformation, exposing a splice site in the *THIC* 3ʹ-UTR. The consequent splicing eliminates the consensus polyadenylation signal and results in unstable long 3ʹ-UTR transcripts, reducing THIC protein levels and subsequently decreasing biosynthesis *de novo* of TPP.

Acquiring data about micronutrient contents in staple crops is essential to understand the potential for exploiting genetic diversity for increased micronutrient content and therefore human health. Different varieties of the same species, as well as wild species, can display considerable variation in micronutrient contents (Bouis and Welch, 2010). The exploitation of natural variation further assists in the identification of markers for candidate genes that control micronutrient accumulation (Conn *et al.*, 2012), as it has been shown for vitamin A in rice (Vallabhaneni *et al.*, 2009; Yan *et al.*, 2010). Diversity of vitamin B_1_ content has so far been analyzed only in rice and potato germplasm (Villareal and Juliano, 1989; Sotelo *et al.*, 1990; Kennedy and Burlingame, 2003; Goyer and Haynes, 2011; Goyer and Sweek, 2011). Characterization of the diversity of vitamin B_1_ accumulation in the germplasm of staple crops could help in the implementation of biofortification approaches to reduce vitamin B_1_ deficiencies. Such deficiencies occur at high frequency in populations whose diets are either poor in sources of vitamin B_1_ or rich in thiaminase, a thiamine-degrading enzyme, which is abundantly present in raw and fermented fish sauce (a common Asian delicacy) as well as certain vegetables and roasted insects consumed primarily in Africa and Asia (Boros, 2000; Barennes *et al.*, 2015).

Here, we report the natural variation of vitamin B_1_ content in 41 cassava accessions grown under controlled conditions, and further investigate vitamin B_1_ biosynthesis and regulation. We quantified the total vitamin B_1_ content in leaves and storage roots by HPLC, and characterized the B_1_ vitamer profiles in both tissues. In order to identify potential determinants of vitamin B_1_ accumulation, we analyzed the transcriptional regulation of genes encoding key enzymes involved in vitamin B_1_ biosynthesis *de novo* in accessions contrasting in vitamin B_1_ content.

## Materials and methods

### Plant material

Cassava accessions were obtained as *in vitro* plantlet material from germplasm collections at ETH Zurich (Swiss Federal Institute of Technology, Switzerland), IITA (International Institute of Tropical Agriculture, Nigeria), CIAT (International Center for Tropical Agriculture, Columbia), MARI (Mikocheni Agricultural Research Institute, Tanzania), and CTCRI (Central Tuber Crops Research Institute, India) (see Supplementary Table S1 at *JXB* online). Each accession was vegetatively propagated *in vitro* on cassava basic medium [CBM: 1× Murashige and Skoog (MS) medium including vitamins (Duchefa), 2% (w/v) sucrose, 2 μM copper(II) sulfate, and 0.3% (w/v) gelrite; pH 5.8] and grown for 1 month in a climate chamber at 28 °C under a 16/8 h light/dark regime. Plantlets were then transferred to soil following a previously described procedure (Bull *et al.*, 2009) and grown under greenhouse conditions (16 h light at 26 °C and 60% humidity, 8 h dark at 17 °C and 50% humidity). Leaves and storage roots from 5-month-old cassava plants were sampled for analysis. Three replicates for each of the 41 cassava accessions were used in the preliminary screening. The confirmation screening on the 18 selected cassava accessions included four biological replicates for each accession. The three youngest fully expanded leaves were sampled (without petioles) from the top of the plants and immediately frozen in liquid nitrogen. The storage roots were washed with water, peeled, and the starchy tissue immediately frozen in liquid nitrogen. To ensure that the moisture content of the tissues did not influence the analyses of vitamin B_1_ content, the dry matter content was calculated on the basis of the mass difference after drying the samples at 40 °C for 1 week.

For the time-course experiment, selected cassava accessions were first propagated *in vitro* and then grown under greenhouse conditions for 7 months following the above-described procedure. Plants were sampled every 4 h for 24 h as well as 1 h before the end of the sunlight period, 1 h before the end of the supplementary artificial light period, and 1 h before the end of the dark period. At each time point, a pool of leaf portions (corresponding to half to one lobe) from four fully expanded apical leaves was sampled and immediately frozen in liquid nitrogen.

For the sequencing of cassava *THIC* genes, cv. 60444 plantlets were vegetatively propagated *in vitro* on CBM without vitamins and grown for 10 days in a climate chamber at 28 °C under a 16/8 h light/dark regime. Plantlets were then transferred to CBM without vitamins, or supplemented with 10 μM of commercial thiamin hydrochloride (Sigma-Aldrich) for 24 h, prior to leaf sampling.

Thermal processing experiments were performed with approximately 30 cm-long commercial waxed cassava roots imported from Costa Rica. They were processed in two different ways for the evaluation of vitamin B_1_: (i) storage roots were peeled, sliced into ~70 g sections, and boiled in 2 l of tap water for 30 min; (ii) storage roots were peeled, sliced into ~70 g sections and soaked in 1 l of tap water for 90 min, then rinsed and boiled in fresh tap water (2 l) for 30 min. The dry matter in each sample was evaluated by drying samples for 5 days at 50 °C.

### Vitamin B_1_ quantification

#### Yeast bioassay

Yeast bioassays for vitamin B_1_ content were performed according to a method previously established with the *thi4* auxotrophic strain of *Saccharomyces cerevisiae* (Raschke *et al.*, 2007). Vitamin B_1_ was extracted from 50 mg of leaves and 100 mg of storage roots. Frozen ground tissues were resuspended in 20 mM sulfuric acid (ratio: 100 mg tissue:1 ml extraction buffer) and incubated at room temperature for 30 min in the dark, and the extract was sterilized at 100 °C for 1 h. After extraction, the solution was adjusted to pH 5.7 using 3 M sodium acetate and centrifuged. The supernatant was then treated with acid phosphatase type I (Sigma) (0.2 U/10 μl in 50 μl plant extract) for 12–15 h at 37 °C to convert the phosphorylated forms of vitamin B_1_ to non-phosphorylated forms. Total vitamin B_1_ content was calculated from the linear range of a dose–response curve established with known amounts of commercial thiamin hydrochloride (Sigma-Aldrich).

#### HPLC measurements

Quantification of B_1_ vitamers was performed according to a previously established HPLC method with minor modifications (Moulin *et al.*, 2013). B_1_ vitamers were extracted from 50 mg of frozen ground tissues using 100 μl of 1% (v/v) trichloroacetic acid. The mixture was vortexed at room temperature for 30 min and centrifuged at 16100 *g* for 10 min at room temperature. The clear supernatant was neutralized by adding 10% of the final volume of 3 M sodium acetate. Samples were oxidized by the addition of 30 mM potassium ferricyanide before separation on a Cosmosil π-NAP column (150 × 4.6 mm, 3 μm pore size). Quantification of TMP, TPP, and thiamin thiochrome derivatives was performed by integrating the corresponding fluorescent peak areas extrapolated from standard curves of similarly treated commercial B_1_ vitamers (TMP chloride, Fluka; TPP chloride, Sigma; thiamin hydrochloride, Fluka). Data were normalized to tissue fresh weight.

### Sequencing and expression analysis of cassava genes from the vitamin B_1_ biosynthesis *de novo* pathway

#### RNA extraction

Total RNA from leaves and storage roots was extracted according to a method previously reported by Cazzonelli *et al.* (1998) with some minor modifications. Approximately 200–300 mg of frozen ground tissues were mixed with 1 ml of lysis buffer [150 mM Tris base adjusted to pH 7.5 with boric acid, containing 2% (w/v) SDS and 50 mM EDTA], vortexed for 5 min, and centrifuged at 16100 *g* for 3 min at room temperature. Absolute ethanol (0.25 volumes) and 5 M potassium acetate (0.11 volumes) were added to 800 μl of the supernatant. The mixture was extracted twice with 1 volume of chloroform:isoamylalcohol (24:1, pH 7.5–8.0) and 1 volume of phenol:chloroform:isoamylalcohol (25:24:1, pH 7.5–8.0). Nucleic acids from the recovered aqueous phase were precipitated in 1 ml of absolute ethanol for 30 min at –80 °C and centrifuged at 16100 *g* for 30 min at 4 °C. The pellet was washed with 80% ethanol and resuspended in 200 μl of diethylpyrocarbonate (DEPC)-treated water. DEPC-treated water was prepared by incubating 0.1% DEPC in distilled water overnight, and subsequently autoclaved. RNA was precipitated with lithium chloride (final concentration 2 M) overnight at 4 °C. The extract was centrifuged for 30 min at 4 °C and the RNA pellet washed with 80% and 100% ethanol, vacuum dried, and resuspended in DEPC-treated water.

#### In silico identification of cassava vitamin B_1_ biosynthetic genes

The cassava orthologs of the Arabidopsis genes encoding vitamin B_1_ biosynthetic enzymes were identified by BLASTing the Arabidopsis protein sequences from the TAIR10 database (Lamesch *et al.*, 2012) against the available translated *Manihot esculenta* v6.1 genome in Phytozome (Prochnik *et al.*, 2012).

#### Amplification and sequencing of MeTHIC1 and MeTHIC2 3ʹ-UTR splice variants

RNA was extracted from leaves using the above-described protocol. cDNA was synthesized with the RevertAid First Strand cDNA Synthesis Kit (Thermo Fisher Scientific AG) according to the manufacturer’s instructions, using 1 μg total RNA. cDNA was synthesized in three different ways: (i) using oligo-(dT)_18_ primers, (ii) using random hexamer oligonucleotides, and (iii) by a 3ʹ-RACE (rapid amplification of cDNA ends) procedure, using oligo-(dT)_18_-adapter primers [GCTGTCAACGATACGCTACGTAACGGCATGACAGTG(T)_18_] taking advantage of the natural poly(A) tail in mRNA as a generic priming site for PCR. For cDNA synthesized with oligo-(dT)_18_ and random hexamer oligonucelotide primers, the 3ʹ-UTR splice variants were amplified by PCR using *MeTHIC1* and *MeTHIC2* specific primer sets. Forward primers were located at the 3ʹ end of *THIC* exon 4 and reverse primers at the end of the 3ʹ-UTR sequence predicted by Phytozome (Prochnik *et al.*, 2012). For cDNA synthesized with oligo-(dT)_18_-adapter primers, the 3ʹ-UTR splice variants were amplified by PCR using *MeTHIC1* and *MeTHIC2* specific forward primers located at the end of *THIC* exon 4 and reverse primers in the adapter region. A first PCR was performed using 3ʹ-RACE primer as the reverse primer and a second one, with the PCR product as template, was performed using 3ʹ-RACE nested primer. PCR products were run on a 1% (w/v) agarose gel and the different bands, corresponding to *THIC* 3ʹ-UTR splice variants, were extracted, ligated into the pJET1.2/blunt vector with the Clone PCR cloning Kit (Thermo Scientific) according to the manufacturer’s instructions, and transformed into thermocompetent *Escherichia coli* cells. Two to six clones were sequenced for each 3ʹ-UTR splicing variant using the Sanger sequencing method. Primer sequences are reported in Supplementary Table S2.

#### Real-time quantitative PCR analysis

cDNA was synthesized using random hexamer oligonucleotide primers with the RevertAid First Strand cDNA Synthesis Kit (Thermo Fisher Scientific AG) according to the manufacturer’s instructions, using 1 μg total RNA for leaves and 500 ng total RNA for storage roots. Real-time quantitative PCR (RT-qPCR) reactions were performed using the LightCycler 480 II system (Roche Diagnostics AG) and Fast SYBR® Green Master Mix (Applied Biosystems, Thermo Fisher Scientific). The reaction mixture contained 1 μl DEPC-treated water, 1 μM of each primer, 5 μl SYBR® Green Master Mix and 2 μl cDNA diluted 10-fold and 3-fold, respectively, for leaves and storage roots. PCR cycling conditions were as follows: initial denaturation at 95 °C for 2 min followed by 40 cycles of denaturation at 95 °C for 10 s, annealing at 60 °C for 20 s, and extension at 72 °C for 30 s. Relative target gene expression levels were normalized to three reference genes, *MePP2A*, *MeUBQ10*, and *MevATPs* (Moreno *et al.*, 2011), averaged by their geometric mean (Vandesompele *et al.*, 2002). Efficiencies of primer pairs were measured and confirmed to be similar, allowing the use of the 2^-ΔΔCT^ method (Livak and Schmittgen, 2001) to calculate relative gene expression levels. Primer sequences are reported in Supplementary Table S3.

### Statistical analysis

The effect of the cultivars on vitamin B_1_ content, gene expression levels, and phenotype was evaluated by one-way analysis of variance (ANOVA) at the 0.05 significance level. When the effect was significant, the ANOVA was followed by a Tukey’s test for *post-hoc* pairwise comparisons (α=0.05). Normality was assessed using the Shapiro-Wilk test on residuals (α=0.01), and homoscedasticity was tested using Bartlett’s test (α=0.01). The gene expression levels in leaves and storage roots of each cultivar were compared using Student’s *t*-test. A bilateral test was applied and type 2 (homoscedasticity) or 3 (hetereoscedasticity) was determined by Fisher’s test of equality of variance (F-test; α=0.05). Expression values of *MeTHIC1*, *MeTHI1a*, and *MeTHI1b* in leaves and underground fresh weight of the confirmation screening of 18 accessions displayed moderate deviation from normality and homoscedasticity, possibly associated with the small sample size.

## Results

### Selection of cassava accessions

We selected 41 cassava accessions for a representative diversity of germplasm from different regions in Africa, Central and South America, and Asia. Cultivars were provided by CIAT (http://ciat.cgiar.org/what-we-do/crop-conservation-and-use/cassava-diversity/) and IITA (http://my.iita.org/accession2/), and included traditional cultivars, improved varieties, and elite breeding lines. Our selection was also based on criteria such as tolerance and susceptibility to viruses and *Xanthomonas axonopodis* pv. *manihotis*. Two additional wild relative species (*Manihot pseudoglaziovii* and *Manihot tristis*) were included in the selection to provide a wider range of genetic diversity (Supplementary Table S1). Cassava plants were grown under controlled conditions in a greenhouse. The range of phenotypic variation in stems, leaves, and storage roots was similar in two independent experiments, and the distribution of the accessions according to their phenotypic characteristics (in particular, plant height, above-ground fresh weight, and underground fresh weight) was nearly identical in two independent experiments (Supplementary Table S4).

### Total vitamin B_1_ content in leaves was stable during the sampling period

In Arabidopsis, vitamin B_1_ biosynthesis *de novo* is regulated by the circadian clock, resulting in a reported significant oscillation of the TMP vitamer detected in a single period (Bocobza *et al.*, 2013). *AtTHIC* transcript analysis also revealed induction of the gene by light (Raschke *et al.*, 2007). To determine whether vitamin B_1_ biosynthesis is regulated diurnally in cassava, we measured vitamin B_1_ levels at 4-hour intervals during a 24-hour period in accessions ARG 13, cv. 60444, and BRA 132, using a yeast bioassay (Raschke *et al.*, 2007). Vitamin B_1_ levels in leaves remained stable during the 24-hour period, except for BRA 132, which showed a small but statistically significant decrease during the dark period (Supplementary Fig. S1). Samples were therefore collected from all 41 cassava accessions between 13.30 and 17.00 h, when vitamin B_1_ levels remained stable in the three control accessions (Supplementary Fig. S1).

### Natural variation of vitamin B_1_ levels in greenhouse-grown cassava accessions

For screening purposes, we first measured vitamin B_1_ content in the 41 accessions selected using a yeast bioassay, which is a cost-effective and high-throughput method. We found statistically significant differences for vitamin B_1_ accumulation in leaf and root tissues between the selected accessions (Supplementary Table S5). Accessions with a vitamin B_1_ content below the 25th percentile of the distribution were considered to be accessions with low vitamin B_1_ content, whereas those with a vitamin content above the 75th percentile were considered to be accessions with high vitamin B_1_ content. Eighteen cassava accessions contrasting in terms of the distribution of vitamin B_1_ content in leaves and storage roots were selected for additional independent measurements (Supplementary Table S6). Most accessions had a similar distribution of vitamin B_1_ content in leaves and storage roots in both independent experiments (Supplementary Tables S5 and S6); however, it should be noted that vitamin B_1_ levels and the extent of the variation differed between the two experiments. The distribution of accessions according to their vitamin B_1_ contents in leaves and storage roots was similar based on either per gram of fresh weight or per gram of dry weight (Supplementary Table S6). Our analysis revealed no correlation between the vitamin B_1_ contents in leaves and storage roots (Supplementary Fig. S2). In addition, no correlation was observed between vitamin B_1_ content and biomass (Supplementary Fig. S3). Combining the two independent measurements, we prioritized eight cassava accessions with low, medium, and high vitamin B_1_ contents in leaves and storage roots for further analysis.

**Fig. 1.**
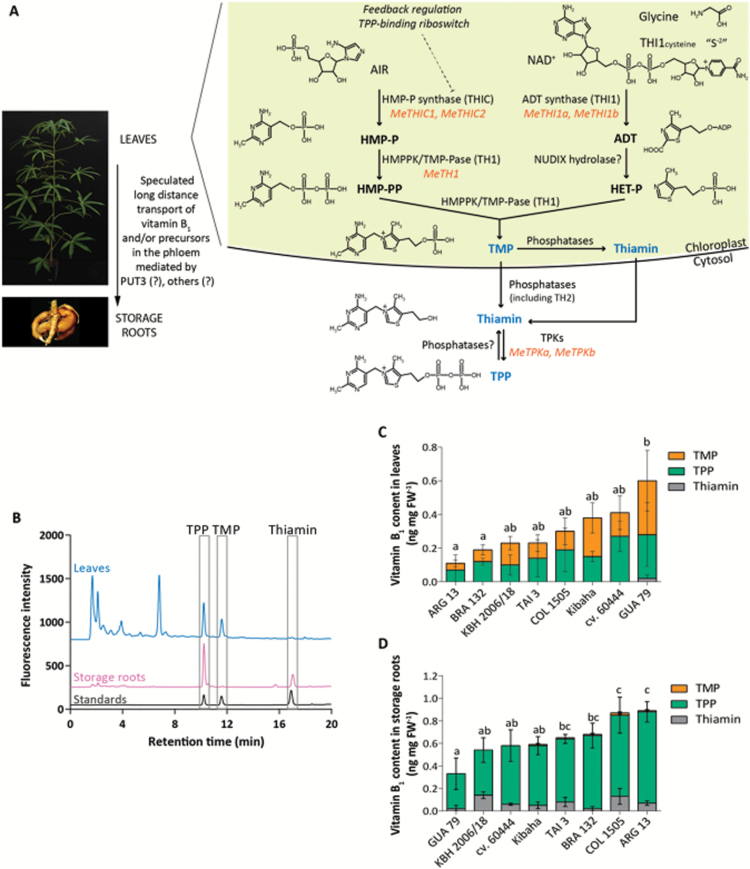
Natural variation of vitamin B_1_ content in selected cassava accessions. (A) Scheme of the vitamin B_1_ biosynthesis pathway as described in Arabidopsis and extrapolated to cassava. The three measured B_1_ vitamers, TMP (thiamin monophosphate), thiamin, and TPP (thiamin pyrophosphate), are shown in blue text. The pyrimidine and thiazole moieties, HMP-P (4-amino-2-methyl-5-hydroxymethylpyrimidine phosphate), HMP-PP (4-amino-2-methyl-5-hydroxymethylpyrimidine pyrophosphate), an adenylated thiazole intermediate (ADT), and HET-P (4-methyl-5-β-hydroxyethylthiazole phosphate), are shown in bold black text. The identified cassava orthologs coding for the known biosynthetic enzymes, HMP-P synthase encoded by *THIC (MeTHIC1*: Manes.02G121700*; MeTHIC2*: Manes.01G164200), ADT synthase encoded by *THI1* (*MeTHI1a*: Manes.15G075600*; MeTHI1b*: Manes.03G123800), HMPPK/TMP-Pase (2-methyl-4-amino-5-hydroxymethylpyrimidine phosphate kinase/thiamin monophosphate pyrophosphorylase) encoded by *TH1* (*MeTH1:* Manes.10G122900), and TPKs (thiamin pyrophosphokinases) encoded by *TPKs (MeTPKa*: Manes.05G063600*; MeTPKb*: Manes.01G217400), are shown in orange italic text. (B) HPLC chromatograms of leaf and storage root extracts. To facilitate visualization, the profile of storage roots was offset by 200 and the profile of leaves was offset by 750 fluorescence units, relative to the baseline. (C, D) HPLC analysis of vitamin B_1_ in (C) leaves and (D) storage roots. Data presented are the mean±SD of four biological replicates. Significant differences (*P*<0.05; Tukey’s multiple comparison test) are indicated by different letters.

We performed HPLC analyses on the eight selected contrasting accessions to provide an accurate quantification of B_1_ vitamers (Fig. 1). Vitamin B_1_ contents determined in the selected accessions were in line with those initially determined using the yeast bioassay, although some discrepancies were observed (Fig. 1C, D; Supplementary Table S6A, B). HPLC analyses confirmed the significant differences of vitamin B_1_ contents in leaves and storage roots, showing a 5.3 (± 3.6)- and a 2.7 (± 0.6)-fold difference between the cassava accessions, respectively (Fig. 1C, D).

### The phosphorylated forms of vitamin B_1_ are the most abundant in cassava and B_1_ vitamer profiles differ in leaves and storage roots

Vitamin B_1_ is mainly present as phosphorylated esters and predominantly as the coenzyme vitamer, TPP, in unicellular organisms (Schweingruber *et al.*, 1991; Moulin *et al.*, 2013) and in leaves of higher plants, including Arabidopsis (Rapala-Kozik *et al.*, 2012; Bocobza *et al.*, 2013; Pourcel *et al.*, 2013) and rice (Dong *et al.*, 2016). Cassava fully expanded leaves also mostly accumulate the phosphorylated forms of vitamin B_1_, with TPP and TMP respectively accounting for 40–66% and 37–58% of total vitamin B_1_ levels. The thiamin vitamer was not detected in most leaf samples (Table 1A). Cassava storage roots mainly accumulated TPP (73–94% of total vitamin B_1_) and thiamin was detected in all accessions (6–26% of total vitamin B_1_), while TMP constituted less than 2% of total vitamin B_1_ (Table 1B).

**Table 1.** HPLC analysis of B_1_ vitamers in leaves and storage roots of selected cassava accessions

A							
Leaves	TPP		TMP		Thiamin		Total vitamin B_1_ (ng mg FW^–1^)
	ng mg FW^–1^	% total vitamin B_1_	ng mg FW^–1^	% total vitamin B_1_	ng mg FW^–1^	% total vitamin B_1_	
ARG 13	0.07 ± 0.09^a^	55 ± 15	0.04 ± 0.02^a^	45 ± 15	nd	nd	0.11 ± 0.11^a^
BRA 132	0.12 ± 0.02^a^	63 ± 8	0.07 ± 0.03^a^	37 ± 8	nd	nd	0.19 ± 0.04^a^
KBH 2006/18	0.10 ± 0.06^a^	44 ± 19	0.13 ± 0.04^ab^	55 ± 19	nd	nd	0.21 ± 0.06^ab^
TAI 3	0.14 ± 0.11^a^	55 ± 27	0.09 ± 0.05^a^	45 ± 27	nd	nd	0.24 ± 0.07^ab^
COL 1505	0.19 ± 0.13^a^	65 ± 6	0.11 ± 0.08^a^	35 ± 6	nd	nd	0.30 ± 0.20^ab^
Kibaha	0.15 ± 0.03^a^	42 ± 13	0.23 ± 0.09^ab^	58 ± 13	nd	nd	0.37 ± 0.10^ab^
cv. 60444	0.27 ± 0.09^a^	66 ± 21	0.14 ± 0.10^ab^	34 ± 21	nd	nd	0.41 ± 0.07^ab^
GUA 79	0.26 ± 0.19^a^	40 ± 8	0.32 ± 0.18^b^	57 ± 9	0.02 ± 0.02	3 ± 3	0.60 ± 0.39^b^

B							
Storage roots	TPP		TMP		Thiamin		Total vitamin B_1_ (ng mg FW^–1^)
	ng mg FW^–1^	% total vitamin B_1_	ng mg FW^–1^	% total vitamin B_1_	ng mg FW^–1^	% total vitamin B_1_	
GUA 79	0.31 ± 0.14^a^	94 ± 5	nd	nd	0.02 ± 0.03^a^	6 ± 5	0.33 ± 0.16^a^
KBH 2006/18	0.40 ± 0.11^ab^	73 ± 9	0.00 ± 0.00^a^	0 ± 1	0.14 ± 0.03^c^	26 ± 8	0.54 ± 0.09^ab^
cv. 60444	0.52 ± 0.14^abc^	90 ± 1	0.00 ± 0.00^a^	0 ± 0	0.06 ± 0.01^ab^	10 ± 1	0.58 ± 0.16^ab^
Kibaha	0.53 ± 0.08^abc^	91 ± 5	0.01 ± 0.01^ab^	1 ± 1	0.05 ± 0.03^a^	8 ± 4	0.59 ± 0.10^ab^
TAI 3	0.56 ± 0.04^abcd^	86 ± 4	0.01 ± 0.00^ab^	2 ± 1	0.08 ± 0.04^abc^	12 ± 5	0.65 ± 0.06^bc^
BRA 132	0.65 ± 0.11^bcd^	95 ± 3	0.01 ± 0.01^ab^	2 ± 1	0.02 ± 0.02^a^	4 ± 3	0.69 ± 0.12^bc^
COL 1505	0.72 ± 0.16^cd^	83 ± 7	0.02 ± 0.01^b^	2 ± 1	0.13 ± 0.07^bc^	15 ± 7	0.87 ± 0.16^c^
ARG 13	0.81 ± 0.09^d^	90 ± 3	0.01 ± 0.01^ab^	2 ± 1	0.07 ± 0.02^abc^	8 ± 3	0.89 ± 0.07^c^

B_1_ vitamer distribution in (A) leaves and (B) storage roots of greenhouse-grown cassava plants. Total vitamin B_1_ content corresponds to the TMP, TPP, and thiamin contents for each replicate. Percentage of total vitamin B_1_ corresponds to the proportion of each vitamer relative to the total vitamin B_1_ content. Data presented are the mean±SD of four biological replicates. Significant differences (*P*<0.05; Tukey’s multiple comparison test) are indicated by different letters. FW, fresh weight; nd, not detected.

### Vitamin B_1_ content in cassava storage roots is not sufficient to reach the dietary recommended daily allowance

**Fig. 2.**
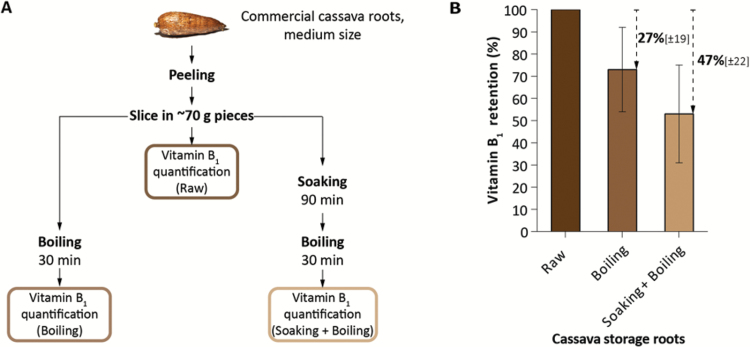
Thermal processing of cassava commercial storage roots and decrease in total vitamin B_1_ retention. (A) Cooking procedures for cassava storage roots. (B) Evaluation of vitamin B_1_ retention in cassava storage roots. Vitamin B_1_ retention was set at 100% for raw cassava; vitamin B_1_ losses following both processing procedures are indicated by the arrows. Vitamin B_1_ was measured using a yeast bioassay. Data presented are the mean±SD of four biological replicates.

It has been reported that 100 g of raw cassava storage root contains 0.087 mg vitamin B_1_ (Fitzpatrick *et al.*, 2012; USDA Nutrient Laboratory, 2016). Based on these data, the recommended daily allowance (RDA) of 1.2 mg day^–1^ vitamin B_1_ for 19- to 30-year-old female adults would be met by consuming 1.4 kg raw cassava. As vitamin B_1_ is known to be heat sensitive and water soluble (Fitzpatrick *et al.*, 2012), losses are expected to occur during the processing and cooking of cassava storage roots. For example, boiling peeled potatoes for 30 minutes leads to a 12% reduction in vitamin B_1_ content (Augustin *et al.*, 1978; Goyer and Haynes, 2011). Therefore, we tested whether processing and cooking alters the vitamin B_1_ content in cassava leaves and storage roots. We used commercial cassava roots and applied thermal processing following typical household processing methods in Northern Mozambique (Muoki and Maziya-Dixon, 2010). Cassava storage roots were peeled, sliced, and boiled in water for 30 min, or soaked for 90 min in standing water and then boiled for 30 min (Fig. 2A). Boiling cassava roots decreased vitamin B_1_ levels by 27 ± 19% and the additional soaking resulted in further substantial losses of up to 47 ± 22% (Fig. 2B; Supplementary Table S7). Our results highlight the importance of measuring vitamin B_1_ in processed and cooked cassava foodstuffs to calculate the recommended daily intake for populations relying mainly on cassava in their diet.

### Vitamin B_1_ biosynthesis *de novo* genes are duplicated in cassava and are expressed at different levels in leaves and storage roots

**Fig. 3.**
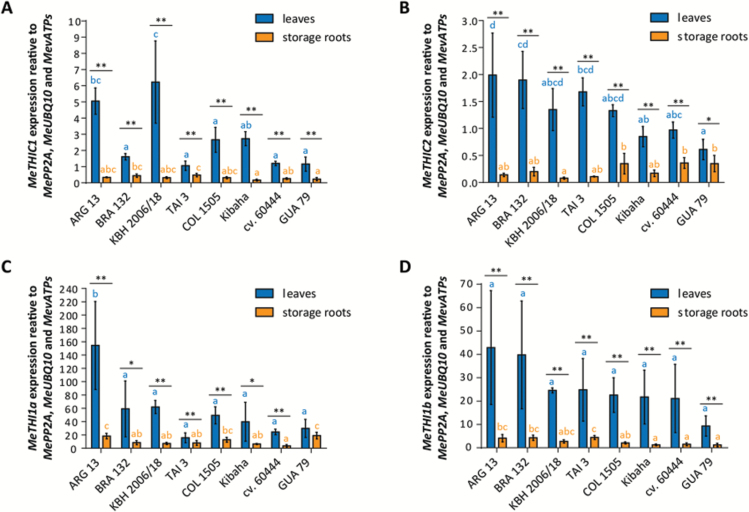
Expression levels of selected vitamin B_1_ biosynthesis *de novo* genes in leaves and storage roots of eight selected cassava accessions. (A) *MeTHIC1*, (B) *MeTHIC2*, (C) *MeTHI1a*, and (D) *MeTHI1b* expression levels in leaves and storage roots of selected cassava accessions contrasting in vitamin B_1_ content in these tissues. Data presented are the mean±SD of four biological replicates [except for leaves: *MeTHI1a* and *MeTHI1b*, TAI 3 (*n*=3) and KBH 2006/18 (*n*=3)]. Significant differences in gene expression levels between accessions in leaves and in storage roots (*P*<0.05; Tukey’s multiple comparison test) are indicated by different letters. Significant differences in gene expression levels between leaves and storage roots in each accession (Student’s *t*-test): *P<0.05, ^**^P<0.01.

In plants, *THIC* and *THI1* genes have been shown to regulate the production *de novo* and levels of vitamin B_1_ in leaves (Pourcel *et al.*, 2013; Dong *et al.*, 2015, 2016; Goyer, 2017). Using the Arabidopsis protein sequence of THIC encoded by the At2g29630 gene as a search parameter in Phytozome (Prochnik *et al.*, 2012), two *THIC* genes were identified in the cassava AM560-2 reference genome v6.1. They were named *MeTHIC1* (Manes.02G121700) and *MeTHIC2* (Manes.01G164200), and the amino acid sequences of the corresponding encoded proteins were 95% identical. Similarly, two cassava *THI1* genes were identified using the Arabidopsis THI1 protein sequence (encoded by At5g54770) as the search parameter, and were named *MeTHI1a* (Manes.15G075600) and *MeTHI1b* (Manes.03G123800). The transcript sequence of the *MeTHI1b* gene appeared to be incomplete in the cassava AM560-2 reference genome. However, the full *MeTHI1b* coding sequence could be determined on the basis of JGI Illumina sequencing data from gDNA for cassava accessions cv. 60444, TMe-3, TMe-7, SC8, and COL 22 (Bredeson *et al.*, 2016) (Supplementary Fig. S4). The amino acid sequences of the corresponding proteins encoded by *MeTHI1a* and *MeTHI1b* appeared to be 94% identical. Despite high similarity at the coding sequence level between the cassava homologs, expression of *MeTHIC1/2* and *MeTHI1a/b* could be discriminated by the design of sequence-specific RT-qPCR primers (Supplementary Table S3). *MeTHIC* and *MeTHI1* genes showed differential expression in the eight selected cassava accessions in both leaves and roots, with the exception of *MeTHI1b* in leaf samples (Fig. 3). Expression was significantly higher in leaves than in storage roots in all of the accessions except for *MeTHI1a* in GUA 79. A similar pattern of expression has been found in Arabidopsis and maize, where *THIC* and *THI1* transcription is high in green tissues and much lower in non-photosynthetic tissues (Guan *et al.*, 2014; Colinas and Fitzpatrick, 2015). Moreover, *MeTHI1* transcripts appeared to be much more abundant than *MeTHIC* transcripts (Fig. 3C, D). THI1 can catalyze only a single turnover and therefore its levels would be expected to be higher than those of THIC, as was also observed in *Chlamydomonas* (Moulin *et al.*, 2013).

### Leaf total vitamin B_1_ content is negatively correlated with expression levels of the *MeTHIC* and *MeTHI1* homologs

**Fig. 4.**
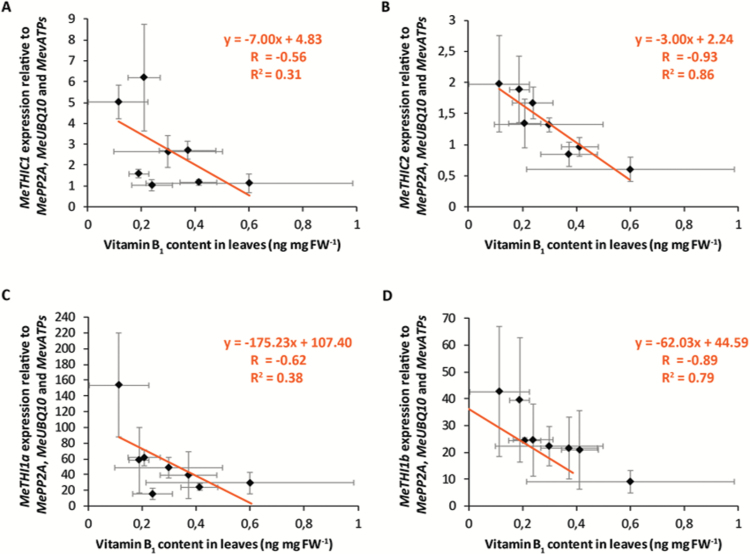
Correlation between vitamin B_1_ content quantified by HPLC and expression levels of selected vitamin B_1_ biosynthetic genes, (A) *MeTHIC1*, (B) *MeTHIC2*, (C) *MeTHI1a*, and (D) *MeTHI1b*, in cassava leaves. Data presented are the mean±SD of four biological replicates for vitamin B_1_ content and for gene expression [except for *MeTHI1a* and *MeTHI1b*, TAI 3 (*n*=3) and KBH 2006/18 (*n*=3)].

Plotting either *MeTHIC1*, *MeTHIC2*, *MeTHI1a*, or *MeTHI1b* gene expression levels in cassava leaves against the vitamin B_1_ content measured by HPLC for each accession revealed a negative correlation in each case (Fig. 4). The strongest negative correlation was observed for *MeTHIC2* (Pearson’s correlation coefficient, *R*= –0.93, *R*^2^=0.86) and *MeTHI1b* (*R*= –0.89, *R*^2^=0.79) (Fig. 4B, D). Our results suggest that the negative correlation between *MeTHIC* transcripts and vitamin B_1_ content could result from the post-transcriptional regulation of *THIC* gene expression mediated by the 3ʹ-UTR riboswitch, as demonstrated in other plant species (Sudarsan *et al.*, 2003; Bocobza *et al.*, 2007, 2013; Wachter *et al.*, 2007). The TPP riboswitch was lost from the *THI1* gene family during gymnosperm evolution (Bocobza *et al.*, 2007), and therefore regulation of *MeTHI1b* transcripts may occur via regulation of *MeTHI1b* promoter activity or alternative post-transcriptional mechanisms.

### *MeTHIC* mRNAs exist as different splice variants

**Fig. 5.**
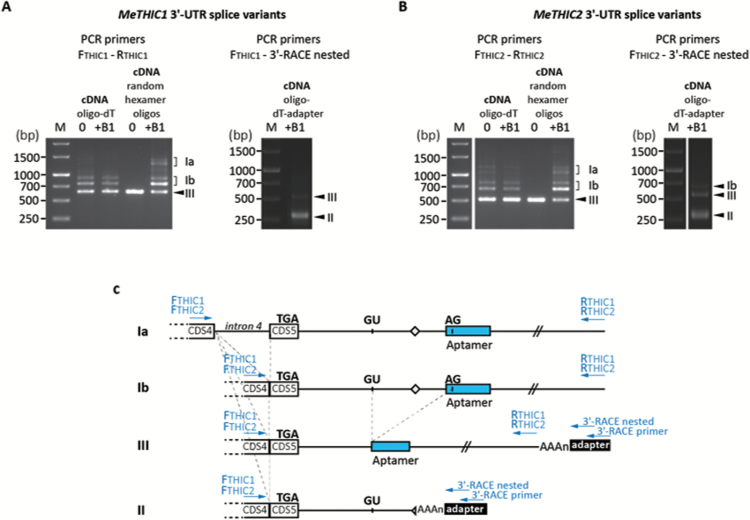
Organization of the cassava *MeTHIC1* and *MeTHIC2* 3ʹ-UTR regions. PCR amplification of (A) *MeTHIC1* and (B) *MeTHIC2* 3ʹ-UTR regions. Plants were grown *in vitro* in cassava basic medium without vitamin B_1_ (indicated by 0) or in medium supplemented with 10 μM of vitamin B_1_ (indicated by +B_1_). cDNA was synthesized using oligo-(dT)_18_ primers, random hexamer oligonucleotides, or oligo-(dT)_18_-adapter primers, and PCR was performed using two different sets of primers for both *MeTHIC* homologs. (C) Organization of the *MeTHIC1* and *MeTHIC2* 3ʹ-UTR regions. CDS4 and CDS5 represent the last exons of the transcripts. An intron (intron 4) is located in front of the stop codon indicated by TGA and is spliced in transcripts Ib, II, and III. GU and AG identify the splice sites to obtain form III and the dashed lines indicate splicing events. The diamond indicates the transcript processing site. Primers used for PCR amplification of *MeTHIC1* and *MeTHIC2* 3ʹ-UTR are indicated by arrows.

Our analysis of the *MeTHIC* homologs revealed that both contain a TPP riboswitch in their respective 3ʹ-UTR, based on sequence homology (Supplementary Fig. S5). *In silico* analysis predicted that the *MeTHIC1* and *MeTHIC2* 3ʹ-UTRs display a structure similar to those predicted for *THIC* 3ʹ-UTRs in other plant species (Wachter *et al.*, 2007). In Arabidopsis, three *THIC* transcript types (I–III) have been identified on the basis of their varying lengths at the 3ʹ-UTR. Type I (*THIC-I*) corresponds to the precursor RNA retaining the complete aptamer. *THIC-I* can either be processed upstream of the aptamer region to result in type II (*THIC-II*, intron-retained variant) with a shorter 3ʹ-UTR, or be spliced into type III (*THIC-III*, intron-spliced variant) with 7 bp missing at the 5ʹ end of the aptamer and a longer 3ʹ-UTR (Wachter *et al.*, 2007). We analyzed *MeTHIC1* and *MeTHIC2* 3ʹ-UTRs in cv. 60444, using cDNA synthesized with oligo-(dT)_18_ primers, random hexamer oligonucleotides, or oligo-(dT)_18_-aptamer primers. We were able to amplify and sequence four 3ʹ-UTR splice variants—*THIC-Ia* retaining intron 4, *THIC-Ib* spliced intron 4 upstream of the stop codon, a *THIC-II* variant, and a *THIC-III* variant—for both *MeTHIC1* and *MeTHIC2* using specific primer pairs (Supplementary Table S2; Fig. 5; Supplementary Fig. S5). PCR primer pairs F_THIC1_-R_THIC1_ (Fig. 5A, left panel) and F_THIC2_-R_THIC2_ (Fig. 5B, left panel) allowed the detection of type I (*MeTHIC1-I*, *MeTHIC2-I*) and the intron-spliced type III (*MeTHIC1-III*, *MeTHIC2-III*) transcripts (Fig. 5C). For both *MeTHIC* homologs, the smallest PCR product corresponds to *MeTHIC-III*, whereas the upper bands correspond to *MeTHIC-Ia* and *MeTHIC-Ib* splicing variants, as well as to products derived from these two splicing forms as previously reported in Arabidopsis (Wachter *et al.*, 2007). The primer pairs F_THIC1_-3ʹ-RACE nested (Fig. 5A, right panel) and F_THIC2_-3ʹ-RACE nested (Fig. 5B, right panel) allow the detection of all three splicing variants if they are polyadenylated. Under these conditions, *MeTHIC1-I* could not be detected (Fig. 5A, right panel) and *MeTHIC2-I* was barely detectable (Fig. 5B, right panel), which suggests that only a minor fraction of the type I transcript is polyadenylated. These results support previous findings in Arabidopsis, where *AtTHIC-I* is likely to be the unprocessed *AtTHIC* pre-mRNA that is further processed and polyadenylated into type II and III mRNA variants (Wachter *et al.*, 2007).

Our analysis revealed that intron 4, which is spliced in *MeTHIC* transcripts Ib, II, and III, is located proximal to the stop codon, similar to what has been reported for *LeTHIC* in tomato (Wachter *et al.*, 2007). This is different from most other analyzed plant species, in which the stop codon is immediately followed by an intron (Wachter *et al.*, 2007). The intron 4 (length in *MeTHIC1*: 493 bp, length in *MeTHIC2*: 366 bp) present in mRNA variant Ia and spliced in Ib, II, and III (Fig. 5C) may represent regular splicing events in cassava because no alternative splice variants II and III retaining this intron could be amplified. However, analysis of *MeTHIC1-Ia* and *MeTHIC2-Ia* transcripts using the ExPASy translate tool (Artimo *et al.*, 2012) revealed an immediate stop codon in intron 4, leading to the translation of THIC1 and THIC2 proteins with two amino acid residue truncations at the C-terminus (Supplementary Fig. S6). It remains to be demonstrated whether this truncation affects THIC functionality.

## Discussion

In this study, we have shown that cassava germplasm has significant variation in vitamin B_1_ accumulation between accessions. Previous studies have shown that vitamin B_1_ content in rice germplasm varies up to 2.8-fold in white rice and up to 2.0-fold in brown rice (Villareal and Juliano, 1989; Sotelo *et al.*, 1990). Similarly, potato germplasm studies, including primitive cultivated wild species, have shown that vitamin B_1_ content varies up to 2.6-fold in mature tubers (Goyer and Sweek, 2011). Our analysis shows a comparable range of variation (i.e. 2.7-fold in storage roots) in the assessed cassava germplasm. Although the 41 cassava accessions we have analyzed represent only a small fraction of the overall genetic diversity in cassava, they are representative of cassava varieties cultivated for subsistence and commercial production (Supplementary Table S1). Our measurements of vitamin B_1_ content in peeled cassava storage roots provide concentration values that are in the range of previous reports (Woot-Tsuen, 1968; Favier, 1977; Montagnac *et al.*, 2009*a*; USDA Nutrient Laboratory, 2016). This suggests that standard greenhouse conditions and a relatively short growth cycle (i.e. 6 months) can be used to estimate vitamin B_1_ in cassava storage roots. However, the average vitamin B_1_ content we measured in cassava leaves was 5- to 5.5-fold below previously reported measurements (Wobeto *et al.*, 2006; Rapala-Kozik *et al.*, 2008, 2012; Shewry *et al.*, 2011). Growing conditions, plant age, and vitamin B_1_ extraction method may explain at least in part these discrepancies.

The comparison of vitamin B_1_ levels in 100 g portions of five major crops shows that raw cassava contains intermediate levels of vitamin B_1_ (Fitzpatrick *et al.*, 2012). The present study has shown that typical processing of cassava roots by soaking and boiling further decreases the amount of vitamin B_1_. Moreover, polyphenolic compounds that accumulate at high levels in cassava (Montagnac *et al.*, 2009*b*) might reduce the bioavailability of vitamin B_1_ by reacting with it to yield non-absorbable vitamin B_1_ disulfide (Hilker and Somogyi, 1982; Hotz and Gibson, 2007; Rapala-Kozik, 2011). Several studies have reported vitamin B_1_ deficiencies in populations whose diet is mostly based on cassava (Adamolekun, 2011). Our HPLC data suggest that genetic variation of vitamin B_1_ content in cassava leaves and storage roots, which was 5.4- and 2.7-fold, respectively (Table 1), could be exploited by trait introgression to address the issue of vitamin B_1_ deficiencies.

We found that total vitamin B_1_ content in cassava leaves has no or only limited variation during a 24-hour diurnal period (Supplementary Fig. S1). Vitamin B_1_ biosynthesis *de novo* in Arabidopsis is regulated by light (Raschke *et al.*, 2007), as well as in a circadian manner via the *THIC* gene promoter (Bocobza *et al.*, 2013). The transcript levels of *AtTHIC* and *AtCCA1*, which encodes the circadian clock regulator CIRCADIAN CLOCK ASSOCIATED 1, oscillate in opposite patterns in Arabidopsis (Bocobza *et al.*, 2013). CCA1 is a transcriptional repressor that can bind to the evening element (AAAATATCT) located 128 bp upstream of the *AtTHIC* 5ʹ-UTR. Our analysis here indicates the absence of CCA1 binding sites in the 1 kb region located upstream of the *MeTHIC1 and MeTHIC2* 5ʹ-UTRs (Supplementary Fig. S7), suggesting that both cassava *THIC* homologs have not retained CCA1-based circadian control of gene expression. However, the absence of circadian regulation remains to be demonstrated for the cassava *THIC* genes.

A direct comparison of the genes involved in the vitamin B_1_ biosynthesis *de novo* pathway in Arabidopsis indicates that the pathway is conserved in cassava, with the notable exception of the duplication of the *THIC* and *THI1* genes. One cassava ortholog (*MeTH1*: Manes.10G122900) of *AtTH1* (At1g22940) and two *TPK* genes (*MeTPKa*: Manes.05G063600 and *MeTPKb*: Manes.01G217400) corresponding to *AtTPK1* (At1g02880) and *AtTPK2* (At2g44750) are present in the cassava genome. The duplication of *THIC* genes is not specific to cassava. Ten of the 64 plant species for which genomes are available in the Phytozome database (Goodstein *et al.*, 2012) have two or more *THIC* genes (Supplementary Fig. S8). In the case of *THI1*, 18 plant species have more than one ortholog (Supplementary Fig. S8). We focused our analysis on *THIC* and *THI1* genes because both were previously shown to regulate the production and level of vitamin B_1_, using either precursor supplementation in Arabidopsis (Pourcel *et al.*, 2013) or transgenic overexpression approaches in Arabidopsis and rice (Dong *et al.*, 2015, 2016; Goyer, 2017). Based on biofortification studies in Arabidopsis and rice, further bottlenecks impeding higher accumulation of thiamin have been hypothesized (Dong *et al.*, 2015; Goyer, 2017).

Sequence analysis of *MeTHIC1* and *MeTHIC2* indicates that both have retained a TPP riboswitch sequence in their 3ʹ-UTRs, with small differences in the P3 stem of the aptamer sequence, which is not directly involved in TPP binding (Wachter *et al.*, 2007) (Supplementary Fig. S5). Expression profiles of *MeTHIC* genes in leaves and roots of selected accessions are similar, although the transcript levels of individual genes can vary (Fig. 3). An alignment of THIC protein sequences shows that the central domain containing the (β/α)_8_ TIM barrel, which likely includes the active site (Coquille *et al.*, 2013), is conserved in both cassava THIC homologs (Supplementary Fig. S9). Moreover, the main residues responsible for binding the AIR substrate, which are located in the iron sulfur cluster binding loop, and the metal ion binding site (Coquille *et al.*, 2013), are conserved in both MeTHIC1 and MeTHIC2 (Supplementary Fig. S9). Collectively, these results suggest that *MeTHIC1* and *MeTHIC2* have retained the same functions. Both MeTHI1a and MeTHI1b share a high similarity with AtTHI1 (Supplementary Fig. S4B) and invariant residues of ADT synthases are conserved in the MeTHI1 proteins (Godoi *et al.*, 2006). MeTHI1 proteins are predicted by TargetP (Emanuelsson *et al.*, 2007) to be targeted to the chloroplast.

Overall, *MeTHIC* and *MeTHI1* transcript levels in the selected accessions were negatively correlated with vitamin B_1_ content in leaves (Fig. 4). The negative feedback regulation of *THIC* mRNAs by the TPP-binding riboswitch has been previously characterized in plants (Bocobza *et al.*, 2007, 2013; Wachter *et al.*, 2007). The strong negative correlation between *MeTHIC2* transcript levels (combined expression of forms Ib, II, and III; Fig. 5) and vitamin B_1_ content (Fig. 4) suggests that a *THIC* riboswitch-based control also regulates vitamin B_1_ content in cassava. The levels of vitamin B_1_ might also be partially feedback-controlled by the regulation of *MeTHI1b*, because transcripts of *MeTHI1b* also have a strong negative correlation with vitamin B_1_ content in cassava leaves (Fig. 4). However, the mechanism regulating *MeTHI1b* transcript levels remains unknown.

The lower accumulation of TMP and the relatively low expression of *MeTHIC* and *MeTHI1* genes in cassava storage roots, compared with leaves, indicate a limited capacity of storage roots to biosynthesize the pyrimidine and thiazole moieties that are the precursors of TMP. However, our results show that cassava storage roots do indeed accumulate vitamin B_1_, which is essential for metabolic activity, including the pentose phosphate pathway, acetyl-CoA biosynthesis, and the tricarboxylic acid cycle (Frank *et al.*, 2007; Rapala-Kozik, 2011). In plants, vitamin B_1_ biosynthesis *de novo* predominantly occurs in green tissues (Guan *et al.*, 2014; Colinas and Fitzpatrick, 2015; Martinis *et al.*, 2016) because most of the biosynthetic enzymes are localized in the chloroplast (Belanger *et al.*, 1995; Chabregas *et al.*, 2001; Ajjawi *et al.*, 2007*b*; Raschke *et al.*, 2007; Kong *et al.*, 2008). In Arabidopsis and maize, vitamin B_1_ biosynthetic genes are expressed strongly in green tissues and to a much lower extent in non-photosynthetic organs, supporting the recently proposed concept of a ‘division of labor’ between photosynthetic and non-photosynthetic tissues, which serve, respectively, as a source and a sink of vitamin B_1_ (Guan *et al.*, 2014; Colinas and Fitzpatrick, 2015). High vitamin B_1_ biosynthesis in leaves and long-distance transport of vitamin B_1_ and/or vitamin B_1_ precursors to sink tissues could compensate for reduced vitamin B_1_ biosynthesis in storage roots to meet the requirements for metabolic activity. A vitamin B_1_ long-distance transporter has recently been identified in Arabidopsis (Martinis *et al.*, 2016). Moreover, thiamin, TMP, and TPP are detectable in phloem sap, implying that these three vitamers could be transported from source leaves to sink organs such as roots (Martinis *et al.*, 2016). The lack of a strong positive correlation between vitamin B_1_ accumulation in cassava leaves and in storage roots (Supplementary Fig. S2) suggests the existence of a complex control of vitamin B_1_ transport and homeostasis. Future studies should focus on the contribution of vitamin B_1_ biosynthesis, salvage, and transport to the accumulation of vitamin B_1_ in cassava organs. This knowledge will help in the implementation of strategies for the development of cassava varieties with enhanced vitamin B_1_ levels in organs/tissues consumed by populations deficient in this micronutrient.

## Supplementary data

Supplementary data are available at *JXB* online.

Fig. S1. Evaluation of vitamin B_1_ content in leaves over a 24 h period.

Fig. S2. Correlation between vitamin B_1_ content in leaves and storage roots.

Fig. S3. Correlation between plant phenotype and vitamin B_1_ content in leaves or storage roots.

Fig. S4. Determination of the *MeTHI1b* full coding sequence and alignment of AtTHI1, MeTHI1a, and MeTHI1b protein sequences.

Fig. S5. Sequenced *MeTHIC1* and *MeTHIC2* 3ʹ-UTR splice variants in the cassava accession cv. 60444.

Fig. S6. Translation of *MeTHIC1* and *MeTHIC2* transcript forms Ia (including intron 4) and Ib (excluding intron 4).

Fig. S7. Analysis for a CCA1 binding motif upstream of the 5ʹ-UTR of Arabidopsis and cassava *THIC* genes.

Fig. S8. Number of *THIC* and *THI1* homologs in the genomes available in the Phytozome database.

Fig. S9. Alignment of THIC protein sequences from Arabidopsis and cassava.

Table S1. Description of the 41 cassava accessions selected for quantification of vitamin B_1_ content.

Table S2. Primers used for PCR amplification of *MeTHIC1* and *MeTHIC2* 3ʹ-UTR splice variants.

Table S3. Primers used for RT-qPCR analysis.

Table S4. Phenotypic characterization of greenhouse-grown cassava accessions.

Table S5. Preliminary quantification of vitamin B_1_ in 41 cassava accessions using a yeast bioassay.

Table S6. Repeated independent quantification of vitamin B_1_ in 18 selected cassava accessions using a yeast bioassay.

Table S7. Thermal processing of cassava commercial storage roots.

## Supplementary Material

Supplementary Figures S1-S9 and Tables S1-S7
