# Supplementary material for: Vitamin B1 diversity and characterization of biosynthesis genes in cassava
Source: J Exp Bot. 2017 Jun 21;68(13):3351–63. doi: 10.1093/jxb/erx196 (PMC5853225; doi:10.1093/jxb/erx196)
Supplement: Supplementary Figures S1-S9 and Tables S1-S7 [file erx196_suppl_supplementary_figures_s1_s9_tables_s1_s7.pdf]

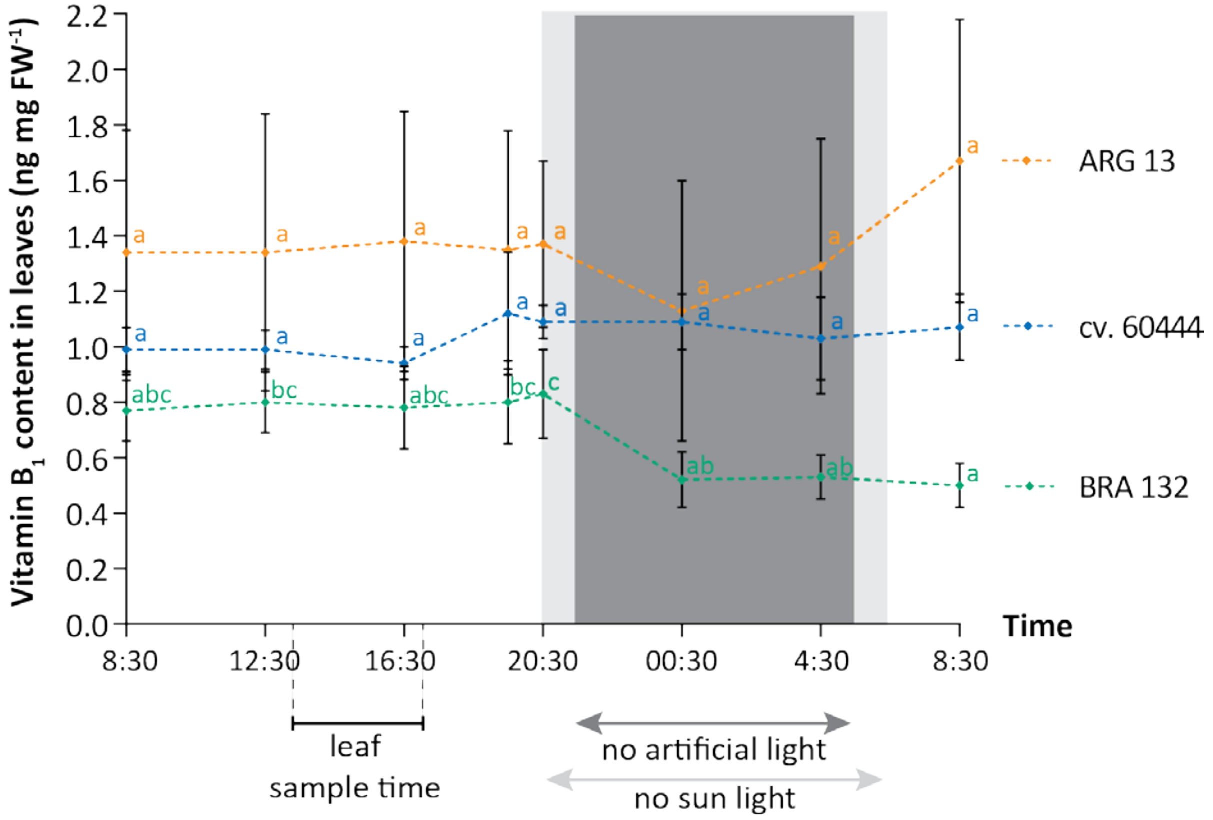

Supplementary Fig. S1. Evaluation of vitamin B_1 content in leaves over a 24 h period.
Leaf samples from 7-month-old greenhouse-grown cassava accessions (BRA 132, cv. 60444 and ARG 13) were collected every 4 h as well as 1 h before the end of the light periods (artificial and sunlight) and 1 h before the end of the dark period. Vitamin B_1 was measured using a yeast bioassay. Average ± S.D. of 4 biological replicates. Tukey's multiple comparison test ($p < 0.05$).

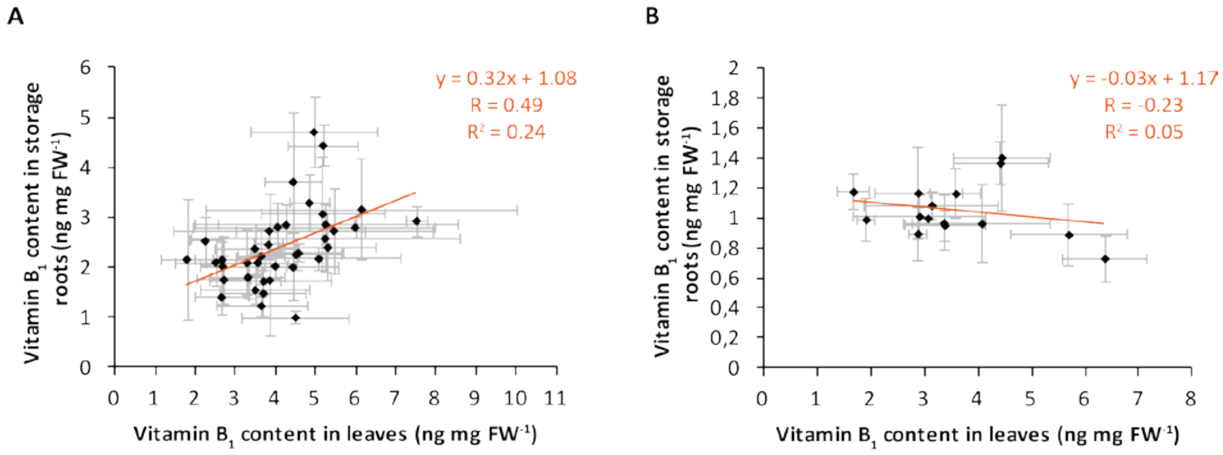

Supplementary Fig. S2. Correlation between vitamin B_1 content in leaves and storage roots.
Vitamin B_1 content in storage roots plotted against vitamin B_1 content in leaves. Vitamin B_1 quantification was performed in 41 cassava accessions **(A)** and repeated in 18 selected accessions independently grown **(B)** using a yeast bioassay. **(A)** Average ± S.D. of 3 biological replicates except TMS 30572 (n=2) for leaves and TMS 30572 (n=2), MEX 95 (n=2), PSE XXX-1 (n=2) for storage roots. T200 is not depicted, as it did not produce storage roots. **(B)** Average ± S.D. of 4 biological replicates except CM2177-2 (n=1), TST-XXX-18 (n=3) for leaves and CM2177-2 (n=1), TST XXX-18 (n=1), MEX 95 (n=3) for storage roots.

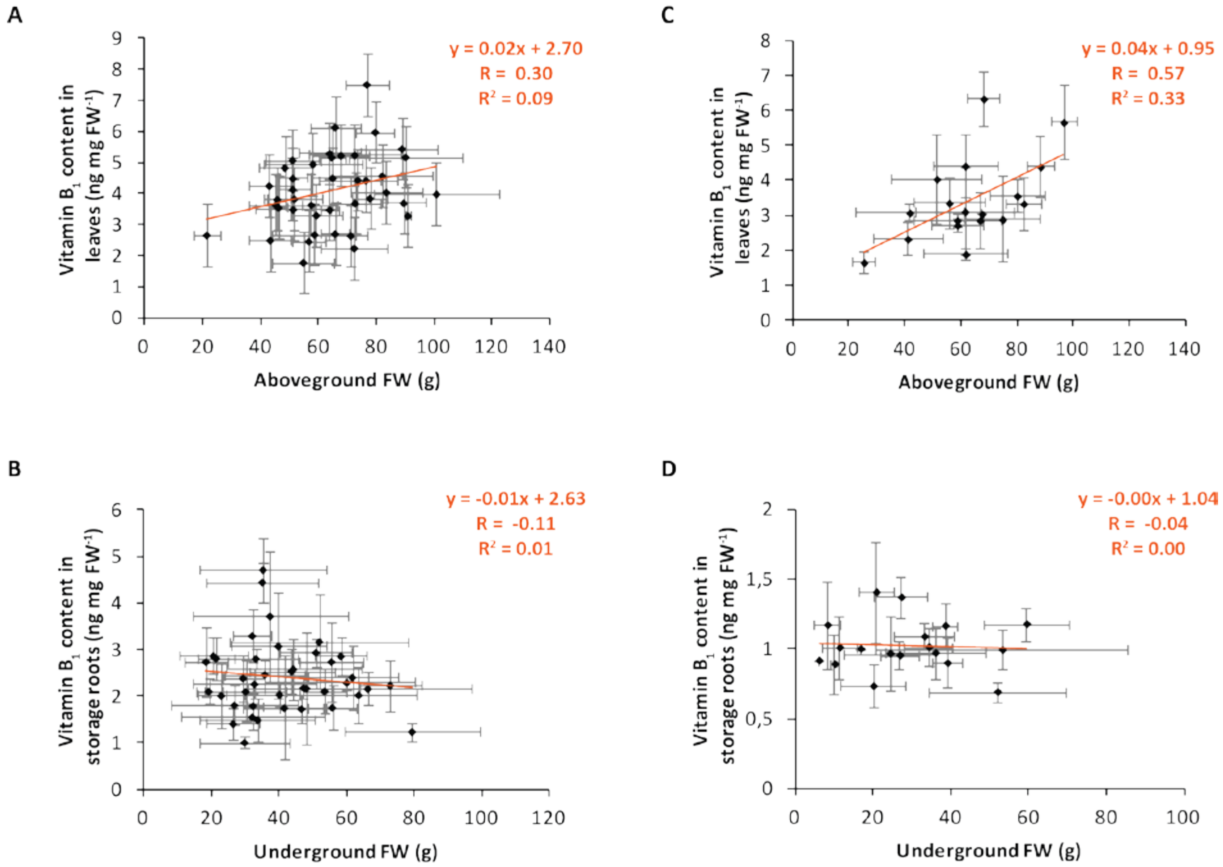

Supplementary Fig. S3. Correlation between plant phenotype and vitamin B_1 content in leaves or storage roots. Vitamin B_1 content in leaves plotted against plant above-ground fresh weight evaluated in 41 cassava accessions **(A)** and repeated in 18 selected accessions grown independently **(C)**. Vitamin B_1 content in storage roots plotted against plant underground fresh weight evaluated in 41 cassava accessions **(B)** and repeated in 18 selected accessions independently grown **(D)**. Vitamin B_1 content was quantified using a yeast bioassay. **(A)** Average ± S.D. of 3 biological replicates except TMS 30572 (n=2). **(B)** Average ± S.D. of 3 biological replicates except TMS 30572 (n=2), MEX 95 (n=2) and PSE XXX-1 (n=2). T200 is not depicted as it did not produce storage roots. **(C)** Average ± S.D. of 4 biological replicates except CM2177-2 (n=1) and TST-XXX-18 (n=3). **(D)** Average ± S.D. of 4 biological replicates except CM2177-2 (n=1), TST XXX-18 (n=1) and MEX 95 (n=3).

A

```
AM560-2_Phytozome     ATGGCCACCATGGCTACAGCTCTCACATCTCTCTCTGCCAAACCCCAGAAGCTCTCTCTC
cv. 60444_JGI         ATGGCCACCATGGCTACAGCTCTCACATCTCTCTCTGCCAAACCCCAGAAGCTCTCTCTC
TMe-3_JGI             ATGGCCACCATGGCTACAGCTCTCACATCTCTCTCTGCCAAACCCCAGAAGCTCTCTCTC
TMe-7_JGI             ATGGCCACCATGGCTACAGCTCTCACATCTCTCTCTGCCAAACCCCAGAAGCTCTCTCTC
SC8_JGI               ATGGCCACCATGGCTACAGCTCTCACATCTCTCTCTGCCAAACCCCAGAAGCTCTCTCTC
COL 22_JGI            ATGGCCACCATGGCTACAGCTCTCACATCTCTCTCTGCCAAACCCCAGAAGCTCTCTCTC
                      ************************************************************

AM560-2_Phytozome     TTTGATTCCTCCGCCTTCTGTGGCACTCCCTTGGCTGCACCCTCCCTTCGCATGCAACCC
cv. 60444_JGI         TTTGATTCCTCCGCCTTCTGTGGCACTCCCTTGGCTGCACCCTCCCTTCGCATGCAACCC
TMe-3_JGI             TTTGATTCCTCCGCCTTCTGTGGCACTCCCTTGGCTGCACCCTCCCTTCGCATGCAACCC
TMe-7_JGI             TTTGATTCCTCCGCCTTCTGTGGCACTCCCTTGGCTGCACCCTCCCTTCGCATGCAACCC
SC8_JGI               TTTGATTCCTCCGCCTTCTGTGGCACTCCCTTGGCTGCACCCTCCCTTCGCATGCAACCC
COL 22_JGI            TTTGATTCCTCCGCCTTCTGTGGCACTCCCTTGGCTGCACCCTCCCTTCGCATGCAACCC
                      ************************************************************

AM560-2_Phytozome     ACGAAAGCTAGCTCTGGTGTATCTGTTTCTATGTCAGCATCTACTCCGCCGCCTTATGAT
cv. 60444_JGI         ACGAAAGCTAGCTCTGGTGTATCTGTTTCTATGTCAGCATCTACTCCGCCGCCTTATGAT
TMe-3_JGI             ACGAAAGCTAGCTCTGGTGTATCTGTTTCTATGTCAGCATCTACTCCGCCGCCTTATGAT
TMe-7_JGI             ACGAAAGCTAGCTCTGGTGTATCTGTTTCTATGTCAGCATCTACTCCGCCGCCTTATGAT
SC8_JGI               ACGAAAGCTAGCTCTGGTGTATCTGTTTCTATGTCAGCATCTACTCCGCCGCCTTATGAT
COL 22_JGI            ACGAAAGCTAGCTCTGGTGTATCTGTTTCTATGTCAGCATCTACTCCGCCGCCTTATGAT
                      ************************************************************

AM560-2_Phytozome     CTGAGTGCTTTCAAGTTTAACCCCATCAAGGAATCCATTGTGGCCCGTGAGATGACGCGC
cv. 60444_JGI         CTGAGTGCTTTCAAGTTTAACCCCATCAAGGAATCCATTGTGGCCCGTGAGATGACGCGC
TMe-3_JGI             CTGAGTGCTTTCAAGTTTAACCCCATCAAGGAATCCATTGTGGCCCGTGAGATGACGCGC
TMe-7_JGI             CTGAGTGCTTTCAAGTTTAACCCCATCAAGGAATCCATTGTGGCCCGTGAGATGACGCGC
SC8_JGI               CTGAGTGCTTTCAAGTTTAACCCCATCAAGGAATCCATTGTGGCCCGTGAGATGACGCGC
COL 22_JGI            CTGAGTGCTTTCAAGTTTAACCCCATCAAGGAATCCATTGTGGCCCGTGAGATGACGCGC
                      ************************************************************

AM560-2_Phytozome     AGGTACATGACGGACATGGTTACGCATGCAGATACTGATGTGGTGATTGTGGGTGCTGGC
cv. 60444_JGI         AGGTACATGACGGACATGGTTACGCATGCAGATACTGATGTGGTGATTGTGGGTGCTGGC
TMe-3_JGI             AGGTACATGACGGACATGGTTACGCATGCAGATACTGATGTGGTGATTGTGGGTGCTGGC
TMe-7_JGI             AGGTACATGACGGACATGGTTACGCATGCAGATACTGATGTGGTGATTGTGGGTGCTGGC
SC8_JGI               AGGTACATGACGGACATGGTTACGCATGCAGATACTGATGTGGTGATTGTGGGTGCTGGC
COL 22_JGI            AGGTACATGACGGACATGGTTACGCATGCAGATACTGATGTGGTGATTGTGGGTGCTGGC
                      ************************************************************

AM560-2_Phytozome     TCTGCTGGCCTCTCTTGTGCTTATGAGCTTAGCAAGAACCCATCTGTGAAGGTTGCCATT
cv. 60444_JGI         TCTGCTGGCCTCTCTTGTGCTTATGAGCTTAGCAAGAACCCATCTGTGAAGGTTGCCATT
TMe-3_JGI             TCTGCTGGCCTCTCTTGTGCTTATGAGCTTAGCAAGAACCCATCTGTGAAGGTTGCCATT
TMe-7_JGI             TCTGCTGGCCTCTCTTGTGCTTATGAGCTTAGCAAGAACCCATCTGTGAAGGTTGCCATT
SC8_JGI               TCTGCTGGCCTCTCTTGTGCTTATGAGCTTAGCAAGAACCCATCTGTGAAGGTTGCCATT
COL 22_JGI            TCTGCTGGCCTCTCTTGTGCTTATGAGCTTAGCAAGAACCCATCTGT-------------
                      ************************************************************

AM560-2_Phytozome     GTTGAGCAATCTGTTAGCCCTGGTGGTGGTGCTTGGCTCGGTGGCCAGCTCTTCTCTGCC
cv. 60444_JGI         GTTGAGCAATCTGTTAGCCCTGGTGGTGGTGCTTGGCTCGGTGGCCAGCTCTTCTCTGCC
TMe-3_JGI             GTTGAGCAATCTGTTAGCCCTGGTGGTGGTGCTTGGCTCGGTGGCCAGCTCTTCTCTGCC
TMe-7_JGI             GTTGAGCAATCTGTTAGCCCTGGTGGTGGTGCTTGGCTCGGTGGCCAGCTCTTCTCTGCC
SC8_JGI               GTTGAGCAATCTGTTAGCCCTGGTGGTGGTGCTTGGCTCGGTGGCCAGCTCTTCTCTGCC
COL 22_JGI            ------------------------------------------------------------
                      ************************************************************

AM560-2_Phytozome     ATG---------------------------------------------------------
cv. 60444_JGI         ATGGTAATCAAAATCCCTTTTTGCAGAGCAGAATCTAAATATTTGTTTCTTTAAAACTGA
TMe-3_JGI             ATGGTAATCAAAATCCCTTTTTGCAGAGCAGAATCTAAATATTTGTTTCTTTAAAACTGA
TMe-7_JGI             ATGGTAATCAAAATCCCTTTTTGCAGAGCAGAATCTAAATATTTGTTTCTTTAAAACTGA
SC8_JGI               ATGGTAATCAAAATCCCTTTTTGCAGAGCAGAATCTAAATATTTGTTTCTTTAAAACTGA
COL 22_JGI            ------------------------------------------------------------
                      ************************************************************

AM560-2_Phytozome     ----------------------------GTGGTTCGCAAACCTGCTCAACTCTTCCTTGA
cv. 60444_JGI         TCTTTCGTATATATGTTTTGTTGGTCAGGTGGTTCGCAAACCTGCTCAACTCTTCCTTGA
TMe-3_JGI             TCTTTCGTATATATGTTTTGTTGGTCAGGTGGTTCGCAAACCTGCTCAACTCTTCCTTGA
TMe-7_JGI             TCTTTCGTATATATGTTTTGTTGGTCAGGTGGTTCGCAAACCTGCTCAACTCTTCCTTGA
SC8_JGI               TCTTTCGTATATATGTTTTGTTGGTCAGGTGGTTCGCAAACCTGCTCAACTCTTCCTTGA
COL 22_JGI            ------------------------------------------------------------
                      ************************************************************

AM560-2_Phytozome     TGAGCTAGGCATCGAGTACGATGAAGCAGACAACTATGTAGTAATCAAGCATGCTGCCCT
cv. 60444_JGI         TGAGCTAGGCATCGAGTACGATGAAGCAGACAACTATGTAGTAATCAAGCATGCTGCCCT
TMe-3_JGI             TGAGCTAGGCATCGAGTACGATGAAGCAGACAACTATGTAGTAATCAAGCATGCTGCCCT
TMe-7_JGI             TGAGCTAGGCATCGAGTACGATGAAGCAGACAACTATGTAGTAATCAAGCATGCTGCCCT
SC8_JGI               TGAGCTAGGCATCGAGTACGATGAAGCAGACAACTATGTAGTAATCAAGCATGCTGCCCT
```

```
COL 22_JGI              ------------------------------------------------------------
                        ************************************************************

AM560-2_Phytozome       GTTCACATCCACAATTATGAGCAAACTCCTAGCCCGCCCCAATGTTAAGCTTTTCAACGC
cv. 60444_JGI           GTTCACATCCACAATTATGAGCAAACTCCTAGCCCGCCCCAATGTTAAGCTTTTCAACGC
TMe-3_JGI               GTTCACATCCACAATTATGAGCAAACTCCTAGCCCGCCCCAATGTTAAGCTTTTCAACGC
TMe-7_JGI               GTTCACATCCACAATTATGAGCAAACTCCTAGCCCGCCCCAATGTTAAGCTTTTCAACGC
SC8_JGI                 GTTCACATCCACAATTATGAGCAAACTCCTAGCCCGCCCCAATGTTAAGCTTTTCAACGC
COL 22_JGI              ------------------------------------------------------------
                        ************************************************************

AM560-2_Phytozome       AGTGGCTGCCGAGGACCTGATAGTGAAGAATGGAAGAGTGGGTGGCGTGGTCACCAACTG
cv. 60444_JGI           AGTGGCTGCCGAGGACCTGATAGTGAAGAATGGAAGAGTGGGTGGCGTGGTCACCAACTG
TMe-3_JGI               AGTGGCTGCCGAGGACCTGATAGTGAAGAATGGAAGAGTGGGTGGCGTGGTCACCAACTG
TMe-7_JGI               AGTGGCTGCCGAGGACCTGATAGTGAAGAATGGAAGAGTGGGTGGCGTGGTCACCAACTG
SC8_JGI                 AGTGGCTGCCGAGGACCTGATAGTGAAGAATGGAAGAGTGGGTGGCGTGGTCACCAACTG
COL 22_JGI              ------------------------------------------------------------
                        ************************************************************

AM560-2_Phytozome       GGCTCTTGTGTCAATGAACCACGACACACAGTCTTGCATGGACCCCAATGTAATGGAGGC
cv. 60444_JGI           GGCTCTTGTGTCAATGAACCACGACACACAGTCTTGCATGGACCCCAATGTAATGGAGGC
TMe-3_JGI               GGCTCTTGTGTCAATGAACCACGACACACAGTCTTGCATGGACCCCAATGTAATGGAGGC
TMe-7_JGI               GGCTCTTGTGTCAATGAACCACGACACACAGTCTTGCATGGACCCCAATGTAATGGAGGC
SC8_JGI                 GGCTCTTGTGTCAATGAACCACGACACACAGTCTTGCATGGACCCCAATGTAATGGAGGC
COL 22_JGI              ------------------------------------------------------------
                        ************************************************************

AM560-2_Phytozome       CAAAGTAGTCGTAAGCTCTTGTGGCCATGATGGACCCTTTGGTGCCACTGGAGTCAAAAG
cv. 60444_JGI           CAAAGTAGTCGTAAGCTCTTGTGGCCATGATGGACCCTTTGGTGCCACTGGAGTCAAAAG
TMe-3_JGI               CAAAGTAGTCGTAAGCTCTTGTGGCCATGATGGACCCTTTGGTGCCACTGGAGTCAAAAG
TMe-7_JGI               CAAAGTAGTCGTAAGCTCTTGTGGCCATGATGGACCCTTTGGTGCCACTGGAGTCAAAAG
SC8_JGI                 CAAAGTAGTCGTAAGCTCTTGTGGCCATGATGGACCCTTTGGTGCCACTGGAGTCAAAAG
COL 22_JGI              ----------------TCTTGTGGCCATGATGGACCCTTTGGTGCCACTGGAGTCAAAAG
                        ************************************************************

AM560-2_Phytozome       GCTGAAGAGCATTGGCATGATTGAGAATGTCCCAGGGATGAAAGCTCTGGACATGAACAC
cv. 60444_JGI           GCTGAAGAGCATTGGCATGATTGAGAATGTCCCAGGGATGAAAGCTCTGGACATGAACAC
TMe-3_JGI               GCTGAAGAGCATTGGCATGATTGAGAATGTCCCAGGGATGAAAGCTCTGGACATGAACAC
TMe-7_JGI               GCTGAAGAGCATTGGCATGATTGAGAATGTCCCAGGGATGAAAGCTCTGGACATGAACAC
SC8_JGI                 GCTAAAGAGCATTGGCATGATTGAGAATGTCCCAGGGATGAAAGCTCTGGACATGAACAC
COL 22_JGI              GCTGAAGAGCATTGGCATGATTGAGAATGTCCCAGGGATGAAAGCTCTGGACATGAACAC
                        ***.********************************************************

AM560-2_Phytozome       TGCTGAAGATGCCATTGTTAGGCTTACCAGAGAGATTGTGCCAGGAATGATTGTCACGGG
cv. 60444_JGI           TGCTGAAGATGCCATTGTTAGGCTTACCAGAGAGATTGTGCCAGGAATGATTGTCACGGG
TMe-3_JGI               TGCTGAAGATGCCATTGTTAGGCTTACCAGAGAGATTGTGCCAGGAATGATTGTCACGGG
TMe-7_JGI               TGCTGAAGATGCCATTGTTAGGCTTACCAGAGAGATTGTGCCAGGAATGATTGTCACGGG
SC8_JGI                 TGCTGAAGATGCCATTGTTAGGCTTACCAGAGAGATTGTGCCAGGAATGATTGTCACGGG
COL 22_JGI              CGCTGAAGATGCCATTGTTAGGCTTACCAGAGAGATTGTGCCAGGAATGATTGTCACTGG
                        .********************************************************.**

AM560-2_Phytozome       CATGGAAGTTGCAGAGATTGATGGCGCTCCAAGAATGGTAGACTTCAATTTCCCTCTTTG
cv. 60444_JGI           CATGGAAGTTGCAGAGATTGATGGCGCTCCAAGAATGGTAGACTTCAATTTCCCTCTTTG
TMe-3_JGI               CATGGAAGTTGCAGAGATTGATGGCGCTCCAAGAATGGTAGACTTCAATTTCCCTCTTTG
TMe-7_JGI               CATGGAAGTTGCAGAGATTGATGGCGCTCCAAGAATGGTAGACTTCAATTTCCCTCTTTG
SC8_JGI                 CATGGAAGTTGCAGAGATTGATGGCGCTCCAAGAATGGTAGACTTCAATTTCCCTCTTTG
COL 22_JGI              CATGGAAGTTGCAGAGATTGATGGCGCTCCAAGAATGGTAGACTTCAATTTCCCTCTTTG
                        ************************************************************

AM560-2_Phytozome       A-----------------------------------------------------------
cv. 60444_JGI           ATGATTAAATTGAACAACTCTAGTGTTTGTAACTGATGAATCCATGCGTGTACTCTGTTG
TMe-3_JGI               ATGATTAAATTGAACAACTCTAGTGTTTGTAACTGATGAATCCATGCGTGTACTCTGTTG
TMe-7_JGI               ATGATTAAATTGAACAACTCTAGTGTTTGTAACTGATGAATCCATGCGTGTACTCTGTTG
SC8_JGI                 ATGATTAAATTGAACAACTCTAGTGTTTGTAACTGATGAATCCATGCGTGTACTCTGTTG
COL 22_JGI              ATGATTAAATTGAACAACTCTAGTGTTTGTAACTGATGAATCCATGCGTGTACTCTGTTG
                        ************************************************************

AM560-2_Phytozome       ------------------------------------------------------------
cv. 60444_JGI           CAGGGACCAACATTTGGAGCCATGATGATATCAGGGCAGAAGGCGGCTCATCTTGCCTTG
TMe-3_JGI               CAGGGACCAACATTTGGAGCCATGATGATATCAGGGCAGAAGGCGGCTCATCTTGCCTTG
TMe-7_JGI               CAGGGACCAACATTTGGAGCCATGATGATATCAGGGCAGAAGGCGGCTCATCTTGCCTTG
SC8_JGI                 CAGGGACCAACATTTGGAGCCATGATGATATCAGGGCAGAAGGCGGCTCATCTTGCCTTG
COL 22_JGI              CAGGGACCAACATTTGGAGCCATGATGATATCAGGGCAGAAGGCGGCTCATCTTGCCTTG
                        ************************************************************
```

```
AM560-2_Phytozome      ------------------------------------------------------------
cv. 60444_JGI          AAGACGCTGGGGCTGCCTAATGCCTTGGATGGAACATACAAGCGAAGCCCAGAATTTGTC
TMe-3_JGI              AAGACGCTGGGGCTGCCTAATGCCTTGGATGGAACATACAAGCGAAGCCCAGAATTTGTC
TMe-7_JGI              AAGACGCTGGGGCTGCCTAATGCCTTGGATGGAACATACAAGCGAAGCCCAGAATTTGTC
SC8_JGI                AAGACGCTGGGGCTGCCTAATGCCTTGGATGGAACATACAAGCGAAGCCCAGAATTTGTC
COL 22_JGI             AAGACGCTGGGGCTGCCTAATGCCTTGGATGGAACATACAAGCGAAGCCCAGAATTTGTC
                       ************************************************************

AM560-2_Phytozome      ---------------------------------------
cv. 60444_JGI          CTAGCAGCAGCAGACTCTGCAGAGACTGCAGATGCTTGA
TMe-3_JGI              CTAGCAGCAGCAGACTCTGCAGAGACTGCAGATGCTTGA
TMe-7_JGI              CTAGCAGCAGCAGACTCTGCAGAGACTGCAGATGCTTGA
SC8_JGI                CTAGCAGCAGCAGACTCTGCAGAGACTGCAGATGCTTGA
COL 22_JGI             CTAGCAGCAGCAGACTCTGCAGAGACTGCAGATGCTTGA
                       ***************************************
```

B

```
AtTHI1          MAAIASTLSLSSTKPQ--RLFDS-SFHGSAISAAPISIGLKP----RS-FSVRATTAGYD
MeTHI1a         MATMATALTSLSAKPQNHSLFDSSAFYGTPVAAPSVRMQPTKSTTAVSISMSASTPPPYN
MeTHI1b         MATMATALTSLSAKPQKLSLFDSSAFCGTPLAAPSLRMQPTKASSGVSVSMSASTPPPYD
                **::*::*:  *:***   **** :* *: ::*  : :  .      *     :*   *:

AtTHI1          LNAFTFDPIKESIVSREMTRRYMTDMITYAETDVVVVGAGSAGLSAAYEISKNPNVQVAI
MeTHI1a         LSAFKFHPIKESIVAREMTRRYMTDMVTHADTDVVIVGAGSAGLSCAYELSKNPSVKVAI
MeTHI1b         LSAFKFNPIKESIVAREMTRRYMTDMVTHADTDVVIVGAGSAGLSCAYELSKNPSVKVAI
                *.**.*.*******:***********:*:*:****:*********.***:****.*:***

AtTHI1          IEQSVSPGGGAWLGGQLFSAMIVRKPAHLFLDEIGVAYDEQDTYVVVKHAALFTSTIMSK
MeTHI1a         VEQSVSPGGGAWLGGQLFSAMVVRKPAHLFLDELGIDYDEADNYVVIKHAALFTSTIMSK
MeTHI1b         VEQSVSPGGGAWLGGQLFSAMVVRKPAQLFLDELGIEYDEADNYVVIKHAALFTSTIMSK
                :********************:*****:*****:*: *** *.***:*************

AtTHI1          LLARPNVKLFNAVAAEDLIVKGNRVGGVVTNWALVAQNHHTQSCMDPNVMEAKIVVSSCG
MeTHI1a         LLARPNVKLFNAVAAEDLIVKNGRVGGVVTNWALVSMNHDTQSCMDPNVMEAKVVVSSCG
MeTHI1b         LLARPNVKLFNAVAAEDLIVKNGRVGGVVTNWALVSMNHDTQSCMDPNVMEAKVVVSSCG
                *********************  ************: **.*************:******

AtTHI1          HDGPFGATGVKRLKSIGMIDHVPGMKALDMNTAEDAIVRLTREVVPGMIVTGMEVAEIDG
MeTHI1a         HDGPFGATGVKRLKSIGMIENVPGMKALDMNTAEDAIVRLTREIVPGMIVTGMEVAEIDG
MeTHI1b         HDGPFGATGVKRLKSIGMIENVPGMKALDMNTAEDAIVRLTREIVPGMIVTGMEVAEIDG
                *******************:.**********************:****************

AtTHI1          APRMGPTFGAMMISGQKAGQLALKALGLPNAIDGTLVGNLSPELVLAAADSAETVDA
MeTHI1a         APRMGPTFGAMMISGQKAAHLALKTLGLPNALEGTFT--LRPEFVIAAADSAETAEA
MeTHI1b         APRMGPTFGAMMISGQKAAHLALKTLGLPNALDGTYK--RSPEFVLAAADSAETADA
                ******************.:****:******::**      **:*:********.:*
```

Supplementary Fig. S4. Determination of the *MeTHI1b* full coding sequence and alignment of AtTHI1, MeTHI1a, MeTHI1b protein sequences.

(A) Determination of the *MeTHI1b* full coding sequence. In the *Manihot esculenta* v6.1 genome available in Phytozome (Prochnik *et al.*, 2012), the *MeTHI1b* coding sequence appears incomplete. To determine the unknown sequence, JGI Illumina sequencing data from gDNA for cassava accessions cv. 60444, TMe-3, TMe-7, SC8 and COL 22 (Bredeson *et al.*, 2016) was assembled into contigs (JGI, personal communication). Consensus sequences were extracted and the *MeTHI1b* truncated coding sequence was used for BLAST searches to identify *MeTHI1b* sequences in each variety. Complete hits with additional 5' and 3' nucleotides were obtained for all varieties, except COL 22 where the internal 449 bp was not contained within assembled contigs. Splice sites were predicted, using the NetPlantGene server (Hebsgaard *et al.*, 1996). An intron was predicted at the 3' end of the truncated *MeTHI1b* gene available in Phytozome, which was not predicted in the annotation. Predicted introns are highlighted in gray. Stars indicate sequence homology between all available sequences. Sequence differences between cultivars are in vermilion. **(B)** Alignment of AtTHI1 (encoded by At5g54770), MeTHI1a (encoded by Manes.15G075600, 78% identity with AtTHI1) and MeTHI1b (encoded by Manes.03G123800, 79% identity with AtTHI1) protein sequences. In gray are highlighted the invariant residues previously hypothesized to play an essential role in the enzymatic activity of THI1 (Godoi *et al.*, 2006). The AtTHI1 protein sequence is from the TAIR10 database (Lamesch *et al.*, 2012), the MeTHI1a protein sequence is from Phytozome (Prochnik *et al.*, 2012) and the MeTHI1b predicted protein sequence were used for the alignment. The translation of the *MeTHI1b* coding sequence predicted from the JGI data **(A)** resulted in a full length protein of high sequence similarity to AtTHI1 **(B)**.

A

***MeTHIC1* (form Ia) – 1308 bp** [F_{THIC1} – R_{THIC1}]

CCGGAAAGTTACATTAGATCTTCTGAAAGGTGAGGATCAAGTTCACTTAAACCTATAAAGGACTTGTACTAATGGTGGAAAGTTTTAGA
ATTGCAATCTGTGGATACGATCTGTAGTGGAAGTACATAATACATTATTTCTAATTGCAACTCCTAAATGCCTAATTTGCCACTTCAACA
TCTATCTAGCAAGATTTAGTGTAACACGGACCAACCGGAGGGAATATAATTTTCTCAGGCGGTTGCTGGCAAGGAATGTTGTCATATG
GGAACGTTGTTATCATATGATCACAGTGCACTCCTATGTCTCTAAAAGATTGTCAACGCAGAAAACTGTAGAGTCTTAACTTATTTTGA
AAATTTTGAATTATTGATGTTAGTCTTTGTTATGCTAATGCTTGACCACCAAGTACCCTTATTGGATTGTGGTTCGCTTACTGCCTGATAT
CATATTTATTTTTAACCAGATGGACTGTCATCGCTAAAAACTTGTAATGAAACGTCTTATTGAATTTTCTTGCAGGAGCATATGAGTGCC
AGGGATTTGGTCAAAAAAGCGTTGGAACTATTATGCATGGTTAGGTTGTGACAATAAGGGATATGCCATTTTGCTTTCTGATAGAGGTAA
GTTGATGGGTTTAGTGTGTTAGGACTGGGAGACATTTTATCTTTGTTCACATCTTGTGTGTCGCCGTAATANANAGGGCAATATTACAATT
GCTACATTCTCTTTATGGGCAAGTTCTTTTTTATCTGGTTGGTTATGTTAACGAAAGAAACGCACCAGGGGTGCCTGTTTTCTGCTTTAATA
CTGGCCATTTGGTCATCTTGAGCAGGTCAGGCTGAGAAAGTCNCTTTGAACCTGAACAGGATAATACCTGCGTAGGGAGTGTGCATTTTC
TTTTCCCTTTTTTGTTCTTGCACAGGGAGGCAGCTTCCTATGCTCGGGCAAGTTGATACACATTGTGCAGATTCACCAAGAATCCTGGGAT
TATTGCTTCAGTGGCTGCAACGTAATCATGGCTGGCAATTCATGCAATTCTTGGATCAATATTTAAAAAAGGGAAGAGAAAATTCGTCAA
ATATTACCATCTATTTTATTTTTGTTTAATACATGGTAAGATGATTCAGAATAATTAAAAATGTCAATTACACAAAATGCGTAGTATATCTG
ATAACAAATAGAAGTGACTATCAAAGACATTAATGATTATAACTTCAGACCCAGCACCCCTCCTCCTGTAAAGGGAACAAATTGACGTTA
AAAATCCTCTTAATGGCCTAAAAGTTAATCCTTTTAATTGCTTGC

***MeTHIC1* (form Ib) – 815 bp** [F_{THIC1} – R_{THIC1}]

CCGGAAAGTTACATTAGATCTTCTGAAAGGAGCATATGAGTGCCAGGGATTTGGTCAAAAAAGCGTTGGAACTATTATGCATGGTTAGG
TTGTGACAATAAGGGATATGCCATTTTGCTNTCTGATAGAGGTAAGTTGATGGGTTTAGTGTGTTAGGACTGGGAGACATTTTATCTTTG
TTCACATCTTGTGTGTCGCCGTAATAAAAAGGGCAATATTACAATTGCTACATTCTCTTTATGGGCAAGTTCTTTTTTATCTGGTTGGTTAT
GTTAACGAAAGAAACGCACCAGGGGTGCCTGTTTTCTGCTTTAATACTGGCCATTTGGTCATCTTGAGCAGGTCAGGCTGAGAAAGTCCC
TTTGAACCTGAACAGGATAATACCTGCGTAGGGAGTGTGCATTTTCTTTTCCCTTTTTTGTTCTTGCACAGGGAGGCAGCTTCCTATGCTC
GGGCAAGTTGATACACATTGTGCAGATTCACCAAGAATCCTGGGATTATTGCTTCAGTGGCTGCAACGTAATCATGGCTGGCAATTCATG
CAATTCTTGGATCAATATTTAAAAAAGGGAAGAGAAAATTCGTCAAATATTACCATCTATTTTATTTTTGTTTAATACATGGTAAGATGATT
CAGAATAATTAAAAATGTCAATTACACAAAATGCGTAGTATATCTGATAACAAATAGAAGTGACTATCAAAGACATTAATGATTATAACTT
CAGACCCAGCACCCCTCCTCCTGTAAAGGGAACAAATTGACGTTAAAAATCCTCTTAATGGCCTAAAAGTTAATCCTTTTAATTGCTTGC

***MeTHIC1* (form III) – 652 bp** [F_{THIC1} – R_{THIC1}]

CCGGAAAGTTACATTAGATCTTCTGAAAGGAGCATATGAGTGCCAGGGATTTGGTCAAAAAAGCGTTGGAACTATTATGCATGGTTAGG
TTGTGACAATAAGGGATATGCCATTTTGCTTTCTGATAGAGGGGTGCCTGTTTTCTGCTTTAATACTGGCCATTTGGTCATCTTGAGCAGG
TCAGGCTGAGAAAGTCCCTTTGAACCTGAACAGGATAATACCTGCGTAGGGAGTGTGCATTTTCTTTTCCCTTTTTTGTTCTTGCACAGGG
AGGCAGCTTCCTATGCTCGGGCAAGTTGATACACATTGTGCAGATTCACCAAGAATCCTGGGATTATTGCTTCAGTGGCTGCAACGTAAT
CATGGCTGGCAATTCATGCAATTCTTGGATCAATATTTAAAAAAGGGAAGAGAAAATTCGTCAAATATTACCATCTATTTTATTTTTGTTTA
ATACATGGTAAGATGATTCAGAATAATTAAAAATGTCAATTACACAAAATGCGTAGTATATCTGATAACAAATAGAAGTGACTATCAAAG
ACATTAATGATTATAACTTCAGACCCAGCACCCCTCCTCCTGTAAAGGGAACAAATTGACGTTAAAAATCCTCTTAATGGCCTAAAAGTTA
ATCCTTTTAATTGCTTGC

***MeTHIC1* (form III) – 517 bp** [F_{THIC1} –3'-RACE nested]

CCGGAAAGTTACATTAGATCTTCTGAAAGGAGCATATGNNNGCCAGGGATTTGGTCAAAAAAGCGTTGGAACTATTATGCATGGTTAGG
TTGTGACAATAAGGGATATGCCATTTTGCTTTCTGATAGAGGGGTGCCTGTTTTCTGCTTTAATACTGGCCATTTGGTCATCTTGAGCAGG
TCAGGCTGAGAAAGTCCCTTTGAACCTGAACAGGATAATACCTGCGTAGGGAGTGTGCATTTTCTTTTCCCTTTTTTGTTCTTGCACAGGG
AGGCAGCTTCCTATGCTCGGGCAAGTTGATACACATTGTGCAGATTCACCAAGAATCCTGGGATTATTGCTTCAGTGGCTGCAACGTAAT
CATGGCTGGCAATTCATGCAATTCTTGGATCAATATTTAAAAAAGGGAAGAGAAAATTCGTCAAATATTACCATCTATTTTATTTTTGTTTA
ATACATGGTAAGATGATTCAGAATAATTAAAAATGTCAATTACACAAAAAAAAAAAAAAAAAAA

***MeTHIC1* (form II) – 275 bp** [F_{THIC1} – 3'-RACE nested]

CCGGAAAGTTACATTAGATCTTCTGAAAGGAGCATATGAGTGCCAGGGATTTGGTCAAAAAAGCGTTGGAACTATTATGCATGGTTAGG
TTGTGACAATAAGGGATATGCCATTTTGCTTTCTGATAGAGGTAAGTTGATGGGTTTAGTGTGTTAGGACTGGGAGACATTTTATCTTTGT
TCACATCTTGTGTGTCGCCGTAATAAAAAGGGCAATATTACAATTGCTACATTCTCTTTATGGGCAAGTTCTTTTTTAAAAAAAAAAAAAA
AAAA

***MeTHIC1* (form II) – 250 bp** [F_{THIC1} – 3'-RACE nested]

CCGGAAAGTTACATTAGATCTTCTGAAAGGAGCATATGAGTGCCAGGGATTTGGTCAAAAAAGCGTTGGAACTATTATGCATGGTTAGG
TTGTGACAATAAGGGATATGCCATTTTGCTTTCTGATAGAGGTAAGTTGATGGGTTTAGTGTGTTAGGACTGGGAGACATTTTATCTTTGT
TCACATCTTGTGTGTCGCCGTAATAAAAAGGGCAATATTACAATTGCTACATTAAAAAAAAAAAAAAAAAA

B

***MeTHIC2* (form Ia) – 1081 bp** [F_{THIC2} – R_{THIC2}]

GCCAGCAAGTTATATTAAATCCTCTAAGAGGTAAGGATCAAATTCAAATAANCCTAAAAAGGTCTTCTACTTTGGTGGAAAATCTAGAA
TTGCATTAAGTAAATAATNCATTAATCATTTGCAATTGCAAGTACACTGCTTACTTAACCACTTTACCATCTGACTAGCAAGATTAAGCA
AAGGGTCCTGTTCCTTGTTATCGTAAAGTCTCCAGGCCAAACTTATTATGGAAGATTGTTTGATATTAGTTTCTGTTATGCTACTGTTTG
AACCATGAAAGTAACCTTGAGATCATGGTTACATTATGCTTGATATCTCATTTGTGTNTNTTATTTGGTTGGACTATGACTTTCCTCACT
TGGAACTTGCAATGTTNTTTGGAATTTTCTTGCAGGAGCATGTGAGGGGGAAGATTTGGTTAAGTCATCATTGGAACTGTGATGCATGT
CTGAGTTGTGAGGATGATGGATATGCTGATTTGCTTTCCAGATGCAGGTAAGTTGATGATTTAGCGTGTTAGGACCTGGAGACATTATCT
TAGTTTGATTCATGTTTGCTGCATTAATAAGAAGCGATAATACAGTTGCTACATTCTCTTTATGTTCAAGTTATTTCTCTCTTTTTTTTTTCTT
TTGTGTGTGTGTATTTATCATTTGGATGTTGTCTTGACGAAAGAAACGCACCAGGGGTGCCTGTATCTGCTTTAATACTGGCCATTTTGGC
CAGAGGATAGAGCTGGTCAGGCTGAGAAAGTCCCTTTGAACCTGAACAGGATAATGCCTGCGTAGGGAGTGTGCATTTTCTTTTCCCTTT
TTCTGTTCTTGCACAGGGAGGCAGCTTCCTATGGCTCGGGCTAGCTGATCCATATGTGCAGAATCAATCGCCATTGAGCCCAGGATTATC
ACTTGAGCGGCTGCAACGTCATCATGGCTGGCAGTTTATGCAATTCTTCAACCAATATTTTAAATAGTAAAGAGAAAATTTGACAAGGCT
TGCTTGATCAGCCATTTATTGGATTTTGGTCAATCTATGGTACGAACNNTAAAATAATAAAAATTTCGTATATCCGATATAACAAGTG

***MeTHIC2* (form Ib) – 717 bp** [F_{THIC2} – R_{THIC2}]

GCCAGCAAGTTATATTAAATCCTCTAAGAGGAGCATGTGAGGGGGAAGATTTGGTTAAGTCATCATTGGAACTGTGATGCATGTCTGAG
TTGTGAGGATGATGGATATGCTGATTTGCTTTCCAGATGCAGGTAAGTTGATGATTTAGCGTGTTAGGACCTGGAGACATTATCTTAGTT
TGATTCATGTTTGCTGCATTAATAAGAAGCGATAATACAGTTGCTACATTCTCTTTATGTTCAAGTTATTTCTCTCTTTTTTTTTTCTTTTGTG
TGTGTGTATTTATCATTTGGATGTTGTCTTGACGAAAGAAACGCACCAGGGGTGCCTGTATCTGCTTTAATACTGGCCATTTTGGCCAGAG
GATAGAGCTGGTCAGGCTGAGAAAGTCCCTTTGAACCTGAACAGGATAATGCCTGCGTAGGGAGTGTGCATTTTCTTTTCCCTTTTTCTGT
TCTTGCACAGGGAGGCAGCTTCCTATGGCTCGGGCTAGCTGATCCATATGTGCAGAATCAATCGCCATTGAGCCCAGGATTATCACTTGA
GCGGCTGCAACGTCATCATGGCTGGCAGTTTATGCAATTCTTCAACCAATATTTTAAATAGTAAAGAGAAAATTTGACAAGGCTTGCTTG
ATCAGCCATTTATTGGATTTTGGTCAATCTATGGTACGAACACTAAAATAATAAAAATTTCGTATATCCGATATAACAAGTG

***MeTHIC2* (form III) – 526 bp** [F_{THIC2} – R_{THIC2}]

GCCAGCAAGTTATATTAAATCCTCTAAGAGGAGCATGTGAGGGGGAAGATTTGGTTAAGTCATCATTGGAACTGTGATGCATGTCTGAG
TTGTGAGGATGATGGATATGCTGATTTGCTTTCCAGATGCAGGGGTGCCTGTATCTGCTTTAATACTGGCCATTTTGGCCAGAGGATAGA
GCTGGTCAGGCTGAGAAAGTCCCTTTGAACCTGAACAGGATAATGCCTGCGTAGGGAGTGTGCATTTTCTTTTCCCTTTTTCTGTTCTTGC
ACAGGGAGGCAGCTTCCTATGGCTCGGGCTAGCTGATCCATATGTGCAGAATCAATCGCCATTGAGCCCAGGATTATCACTTGAGCGGC
TGCAACGTCATCATGGCTGGCAGTTTATGCAATTCTTCAACCAATATTTTAAATAGTAAAGAGAAAATTTGACAAGGCTTGCTTGATCAGC
CATTTATTGGATTTTGGTCAATCCATGGTACGATCACTAAAATAATAAAAATTTCGTATATCCGATATAACAAGTG

***MeTHIC2* (form III) – 534 bp** [F_{THIC2} – 3'-RACE nested]

GCCAGCAAGTTATATTAAATCCTCTAAGAGGAGCATGTGAGGGGGAAGATTTGGTTAAGTCATCATTGGAACTGTGATGCATGTCTGAG
TTGTGAGGATGATGGATATGCTGATTTGCTTTCCAGATGCAGGGGTGCCTGTATCTGCTTTAATACTGGCCATTTTGGCCAGAGGATAGA
GCTGGTCAGGCTGAGAAAGTCCCTTTGAACCTGAACAGGATAATGCCTGCGTAGGGAGTGTGCATTTTCTTTTCCCTTTTTCTGTTCTTGC
ACAGGGAGGCAGCTTCCTATGGCTCGGGCTAGCTGATCCATATGTGCAGAATCAATCGCCATTGAGCCCAGGATTATCACTTGAGCGGC
TGCAACGTCATCATGGCTGGCAGTTTATGCAATTCTTCAACCAATATTTTAAATAGTAAAGAGAAAATTTGACAAGGCTTGCTTGATCAGC
CATTTATTGGATTTTGGTCAATCTATGGTACGAACACTAAAATAATAAAAATTTCGTATATCCGATAAAAAAAAAAAAAAAAAA

***MeTHIC2* (form III) – 553 bp** [F_{THIC2} – 3'-RACE nested]

GCCAGCAAGTTATATTAAATCCTCTAAGAGGAGCATGTGAGGGGGAAGATTTGGTTAAGTCATCATTGGAACTGTGATGCATGTCTGAG
TTGTGAGGATGATGGATATGCTGATTTGCTTTCCAGATGCAGGGGTGCCTGTATCTGCTTTAATACTGGCCATTTTGGCCAGAGGATAGA
GCTGGTCAGGCTGAGAAAGTCCCTTTGAACCTGAACAGGATAATGCCTGCGTAGGGAGTGTGCATTTTCTTTTCCCTTTTTCTGTTCTTGC
ACAGGGAGGCAGCTTCCTATGGCTCGGGCTAGCTGATCCATATGTGCAGAATCNATCGCCATTGAGCCCAGGATTATCACTTGAGCGGC

TGCAACGTCATCATGGCTGGCAGTTTATGCAATTCTTCAACCAATATTTTAAATAGTAAAGAGAAAATTTGANNAGGCTTGCTTGATCAG
CCATTTATTGGATTTTGGTCAATNNATGGTACGAANNNNAAAANAANNAAAATTTCGTATATCCGATNTANNNAGTGANNCNNNNNN
NNNNNAAAAAAAAAAAAA

MeTHIC2 (form II) – 281 bp [F_{THIC2} – 3'-RACE nested]
GCCAGCAAGTTATATTAAATCCTCTAAGAGGAGCATGTGAGGGGGAAGATTTGGTTAAGTCATCATTGGAACTGTGATGCATGTCTGAG
TTGTGAGGATGATGGATATGCTGATTTGCTTTCCAGATGCAGGTAAGTTGATGATTTAGCGTGTTAGGACCTGGAGACATTATCTTAGTT
TGATTCATGTTTGCTGCATTAATAAGAAGCGATAATACAGTTGCTACATTCTCTTTATGTTCAAGTTATTTCTCTCNNNNNNNTTTAAAAA
AAAAAAAAAAA

C

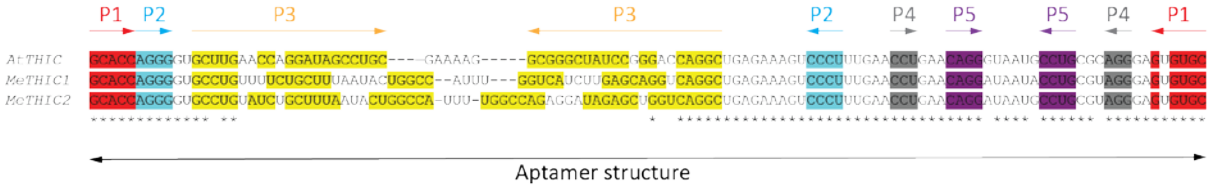

Supplementary Fig. S5. Sequenced _MeTHIC1_ and _MeTHIC2_ 3'-UTR splice variants in the cassava accession cv. 60444.
Sequences of the *MeTHIC1* **(A)** and *MeTHIC2* **(B)** 3'-UTR splice variants amplified from different cDNA templates and using the primers indicated in brackets. Primer sequences or polyA are underlined. The stop codon is indicated by TGA. The intron sequenced in splice form Ia is represented in bold gray. The full-length aptamer is highlighted in yellow and the truncated aptamer present in form III is highlighted in blue. GT and AG represent the splice sites to generate form III. **(C)** Alignment of Arabidopsis and cassava *THIC* 3'-UTR aptamer structures. The asterisks indicate the nucleotides conserved between *AtTHIC*, *MeTHIC1* and *MeTHIC2*. The nucleotides forming the five stems (P1 to P5) are highlighted in color.

A. *MeTHIC1* (Manes.02G121700)

> Transcript sequence including intron 4

ATGGCGTCTGTGCAAGCTACTAGTTTTGCCTCATCTGTGTGCAAAAATGGCACCCAGAAGTTTCCGAGCAGCTCCTTCTTGCCGGGTTTTGATGTCGTGGCT
GGCCGTGGATTTATTAAGAAGGAAACTTTACCTCGCTCTTTGATCTCATCTATTCCCAGAGCCACCTTAACCTTTGATCCTCCAACTACCAATTCAGATAAAA
TTAAACAGAGGAAGCATACTCTTGATCCTTCTTCTCCTGATTTTCTTCCTCTTCCCTCTTTCGAACAGTGTTTTCCAAGGAGTACAAAAGAATACAGGGAAGT
TGTTCATGAACAGACTGGCCATGTTCTCAAGGTTCCTTTCAGGCGGGTCCACTTGTCTGGGGATGAACCCTGTTTCGATAATTATGATACCAGTGGTCCTCA
AAACATTAGTCCCCGAACAGGACTTCCTAAACTGCGTAAAGACTGGGTTGATAGGCGGGAGAAGCTAGGCACACCAAGATACACTCAGATGTACTATGCG
AAGCAAGGAATAATAACTGAGGAAATGCTGTATTGTGCTGTTCGAGAGAAACTTGACCCAGAGTTTGTGAGGTCAGAGGTTGCTCGTGGACGGGCAATAA
TCCCTTCCAACAAGAAGCACCTAGAATTACAGCCAATGATAGTTGGAAGAAATTTTTTGGTCAAAGTCAATGCTAATATTGGAAATTCTGCTGTTGCAAGCT
CTATTGAGGAAGAAGTTTATAAGGTTCAATGGGCAACCATGTGGGGTGCTGACACTGTCATGGACCTTTCAACAGGTCGCCACATTCACGAGACCCGTGAA
TGGATATTACGAAACTCTGCTGTGCCAGTTGGAACTGTGCCCATCTATCAAGCACTTGAGAAAGTTAATGGAATCGCTGAAAATCTGAGCTGGGAAGTCTT
CAGAGAGACATTGATTGAACAAGCCGAGCAAGGAGTAGATTATTTCACTATCCATGCTGGGGTTCTTCTGCGATACATTCCTTTAACTGCAAAACGAATGA
CAGGAATTGTTTCTCGTGGAGGGTCAATTCATGCCAAGTGGTGCTTGGCTTATCACAAGGAAAATTTTGCTTATGAACACTGGGATGACATACTTGACATCT
GTAATCAATATGATGTGGCCCTTTCAATTGGTGATGGGCTGAGACCTGGGTCCATATATGATGCAAATGACACTGCACAATTTGCAGAGCTCTTGACTCAG
GGGGAGCTAACCCGTCGAGCATGGGAGAAGGACGTGCAGGTAATGAATGAAGGACCTGGCCATATTCCTATGCACAAAATCCCAGAGAACATGCAAAAA
CAGCTAGAGTGGTGCAATGAAGCACCTTTCTACACTCTTGGACCCCTGACAACTGATATTGCTCCCGGATACGATCACATTACCTCTGCCATTGGTGCTGCC
AACATCGGGGCTCTAGGCACTGCACTCCTCTGTTATGTCACACCAAAGGAGCATCTTGGGTTACCAAATAGGGATGATGTGAAGGCTGGAGTTATAGCATA
TAAGATATCTGCACATGCAGCTGATTTAGCAAAAGGTCACCCTCACGCTCAAGCCTGGGATGATGCATTAAGCAAGGCCCGATTTGAGTTCAGATGGATGG
ACCAATTTGCTTTATCCTTGGACCCGATGACAGCAATGTCCTTCCATGACGAAACCCTGCCATCAGAAGGTGCAAAAGTAGCACATTTTTGCTCTATGTGTG
GTCCAAAGTTCTGCTCCATGAAGATAACAGAGGATGTGAGGAAGTACGCAGAGGAGCATGGTTATGGTAGCGCAGAGGAAGCTGTACAACATGGGATGG
ATGCTATGAGTGCTGAATTCCTGGCTGCTAAGAAAACTGTTAGCGGAGAACAACATGGTGAAGTTGGTGGAGAAATCTACCTGCCGGAAAGTTACATTAG
ATCTTCTGAAAGGTGAGGATCAAGTTCACTTAAACCTATAAAGGACTTGTACTAATGGTGGAAAGTTTTAGAATTGCAATCTGTGGATACGATCTGTAGTG
GAAGTACATAATACATTATTTCTAATTGCAACTCCTAAATGCCTAATTTGCCACTTCAACATCTATCTAGCAAGATTTAGTGTAACACGGACCAACCGGAGG
GAATATAATTTTCTCAGGCGGTTGCTGGCAAGGAATGTTGTCATATGGGAACGTTGTTATCATATGATCACAGTGCACTCCTATGTCTCTAAAAGATTGTCA
ACGCAGAAAACTGTAGAGTCTTAACTTATTTTGAAAATTTTGAATTATTGATGTTAGTCTTTGTTATGCTAATGCTTGACCACCAAGTACCCTTATTGGATTGT
GGTTCGCTTACTGCCTGATATCATATTTATTTTTAACCAGATGGACTGTCATCGCTAAAAACTTGTAATGAAACGTCTTATTGAATTTTCTTGCAGGAGCATA
TGA

> Translation from *MeTHIC1* transcript sequence excluding intron 4

Met A S V Q A T S F A S S V C K N G T Q K F P S S S F L P G F D V V A G R G F I K K E T L P R S L I S S I P R A T L T F D P P T T N S D K I K Q R K H T L
D P S S P D F L P L P S F E Q C F P R S T K E Y R E V V H E Q T G H V L K V P F R R V H L S G D E P C F D N Y D T S G P Q N I S P R T G L P K L R K D
W V D R R E K L G T P R Y T Q **Met** Y Y A K Q G I I T E E **Met** L Y C A V R E K L D P E F V R S E V A R G R A I I P S N K K H L E L Q P **Met** I V G R N F
L V K V N A N I G N S A V A S S I E E E V Y K V Q W A T **Met** W G A D T V **Met** D L S T G R H I H E T R E W I L R N S A V P V G T V P I Y Q A L E K V
N G I A E N L S W E V F R E T L I E Q A E Q G V D Y F T I H A G V L L R Y I P L T A K R **Met** T G I V S R G G S I H A K W C L A Y H K E N F A Y E H W D
D I L D I C N Q Y D V A L S I G D G L R P G S I Y D A N D T A Q F A E L L T Q G E L T R R A W E K D V Q V **Met** N E G P G H I P **Met** H K I P E N **Met**
Q K Q L E W C N E A P F Y T L G P L T T D I A P G Y D H I T S A I G A A N I G A L G T A L L C Y V T P K E H L G L P N R D D V K A G V I A Y K I S A H A
A D L A K G H P H A Q A W D D A L S K A R F E F R W **Met** D Q F A L S L D P **Met** T A **Met** S F H D E T L P S E G A K V A H F C S **Met** C G P K F C S
Met K I T E D V R K Y A E E H G Y G S A E E A V Q H G **Met** D A **Met** S A E F L A A K K T V S G E Q H G E V G G E I Y L P E S Y I R S S E R S I **Stop**

> Translation from *MeTHIC1* transcript sequence including intron 4

Met A S V Q A T S F A S S V C K N G T Q K F P S S S F L P G F D V V A G R G F I K K E T L P R S L I S S I P R A T L T F D P P T T N S D K I K Q R K H T L
D P S S P D F L P L P S F E Q C F P R S T K E Y R E V V H E Q T G H V L K V P F R R V H L S G D E P C F D N Y D T S G P Q N I S P R T G L P K L R K D
W V D R R E K L G T P R Y T Q **Met** Y Y A K Q G I I T E E **Met** L Y C A V R E K L D P E F V R S E V A R G R A I I P S N K K H L E L Q P **Met** I V G R N F
L V K V N A N I G N S A V A S S I E E E V Y K V Q W A T **Met** W G A D T V **Met** D L S T G R H I H E T R E W I L R N S A V P V G T V P I Y Q A L E K V
N G I A E N L S W E V F R E T L I E Q A E Q G V D Y F T I H A G V L L R Y I P L T A K R **Met** T G I V S R G G S I H A K W C L A Y H K E N F A Y E H W D
D I L D I C N Q Y D V A L S I G D G L R P G S I Y D A N D T A Q F A E L L T Q G E L T R R A W E K D V Q V **Met** N E G P G H I P **Met** H K I P E N **Met**
Q K Q L E W C N E A P F Y T L G P L T T D I A P G Y D H I T S A I G A A N I G A L G T A L L C Y V T P K E H L G L P N R D D V K A G V I A Y K I S A H A
A D L A K G H P H A Q A W D D A L S K A R F E F R W **Met** D Q F A L S L D P **Met** T A **Met** S F H D E T L P S E G A K V A H F C S **Met** C G P K F C S
Met K I T E D V R K Y A E E H G Y G S A E E A V Q H G **Met** D A **Met** S A E F L A A K K T V S G E Q H G E V G G E I Y L P E S Y I R S S E R **Stop** G S S
S L K P I K D L Y **Stop** W W K V L E L Q S V D T I C S G S T **Stop** Y I I S N C N S **Stop** **Met** P N L P L Q H L S S K I **Stop** C N T D Q P E G I **Stop** F S Q A
V A G K E C C H **Met** G T L L S Y D H S A L L C L **Stop** K I V N A E N C R V L T Y F E N F E L L **Met** L V F V **Met** L **Met** L D H Q V P L L D C G S L T A
Stop Y H I Y F **Stop** P D G L S S L K T C N E T S Y **Stop** I F L Q E H **Met**

B. MeTHIC2 (Manes.01G164200)

> Transcript sequence including intron 4

ATGGCATCGGTGCAAGCTACTAGTTTGTCCTCTGCTGTGTGCAAAAATGGAAGCCATAAGTTTCCAAGCGGCTCCTTCTTGCCGGGGTTTGATGGTGTGCTT
GGGCGTGGATCTCTTAAGAAGGAAATATGGCCTCGTTCTTTGGCGACATCTGTTCCTAGGGCCACCTTAACCTTTGATCCTCCAACAACAAATTCAGATAAA
GCTAAGCAGAGGAAACATACTGTTGATCCTTCTTCTCCTGATTTCCTTCCTCTTCCCTCCTTCGAACAATGTTTTCCAAGAAGCACCAAAGAATACAGGGAAA
TTGTTCATGAAGAATCCGGTCACGTCCTCAAAGTTCCTTTCAGACGGGTCCACTTGTCTGGGGATGAACCCAGTTTTGATAATTATGATACCAGTGGTCCTC

AAAACATTAGTCCCCGTATCGGAATTCCTAAACTGAGGAAAGACTGGGTTGATAGGCGTGAGAAGCTAGGCGCACCAAGATACAGTCAGATGTACTATGC
GAAGCAAGGAATAATAACAGAGGAAATGTTATATTGTGCTGCTCGTGAGAAGCTTGACCCAGAGTTTGTCAGGTCAGAGGTTGCTCGTGGACGGGCAATA
ATCCCTTCGAACAAGAAGCACCTGGAGTTGGAGCCAATGATTGTTGGAAGAAATTTTTTGGTCAAAGTCAATGCAAATATTGGAAATTCTGCTGTGGCAAG
CTCTATTGAGGAAGAAGTTTATAAGGTTCAATGGGCAACCATGTGGGGTGCTGACACTGTCATGGACCTCTCAACTGGTCGGCACATCCATGAGACCCGTG
AGTGGATCTTACGAAACTCTGCTGTACCGGTTGGAACTGTGCCCATCTATCAAGCACTTGAAAAAGTTAATGGAATTGCTGAAAATCTGAGCTGGGAAGTC
TTCAGAGACACATTGATTGAACAAGCTGAGCAGGGTGTAGACTATTTCACTATCCATGCTGGGGTTCTTCTACGATACATTCCTTTAACTGCTAAACGCATG
ACAGGAATTGTTTCTCGTGGAGGATCAATTCATGCAAAGTGGTGCTTAGCTTATCACAAGGAGAATTTTGCATATGAACACTGGGATGACATACTTGACAT
CTGTAATCAATATGATGTGGCACTTTCGATCGGTGATGGGCTGAGACCTGGGTCCATATATGATGCCAATGACACTGCTCAGTTTGCAGAGCTCTTGACTCA
AGGGGAACTAACCCGTAGAGCATGGGAGAAGGATGTACAGGTGATGAATGAAGGACCTGGCCATATTCCTATGCACAAAATCCCAGAGAACATGCAAAA
ACAGCTCGAATGGTGCAATGAAGCACCTTTCTACACTCTTGGACCCCTGACAACTGATATTGCTCCTGGATATGATCACATTACCTCTGCCATTGGTGCTGCC
AACATTGGGGCTCTTGGCACTGCACTTCTCTGTTATGTCACACCAAAGGAGCATCTTGGGTTGCCAAATAGGGATGATGTGAAGGCAGGTGTTATAGCATA
TAAGATATCTGCACATGCAGCTGATTTAGCAAAAGGTCACCCTCATGCTCAAGCCTGGGATGATGCATTAAGCAAGGCCCGATTTGAGTTCAGATGGATGG
ACCAATTTGCTTTATCCTTGGACCCCATGACTGCAATGTCCTTCCATGATGAAACCCTGCCATCAGAAGGTGCAAAAGTAGCACATTTTTGCTCCATGTGTGG
GCCGAAGTTCTGCTCTATGAAGATAACAGAGGATGTGCGAAAGTATGCGGAAGAGCATGGTTATGGTAGTGCAGAGGAAGCTGTGCAGCACGGGATGG
ATGCCATGAGTGCTGAGTTCCTGGCTGCTAAGAAAACTGTTAGTGGAGAACAACATGGTGAAGTTGGTGGGGAAATCTACCTGCCAGCAAGTTATATTAA
ATCCTCTAAGAGGTAAGGATCAAATTCAAATAAACCTAAAAAGGTCTTCTACTTTGGTGGAAAATCTAGAATTGCATTAAGTAAATAATACATTAATCATTT
GCAATTGCAAGTACACTGCTTACTTAACCACTTTACCATCTGACTAGCAAGATTAAGCAAAGGGTCCTGTTCCTTGTTATCGTAAAGTCTCCAGGCCAAACTT
ATTATGGAAGATTGTTTGATATTAGTTTCTGTTATGCTACTGTTTGAACCATGAAAGTAACCTTGTGAGATCATGGTTACATTATGCTTGATATCTCATTTGT
GTTTTTTATTTGGTTGGACTATGACTTTCCTCACTTGGAACTTGCAATTTTTTTTGGAATTTTCTTGCAGGAGCATGTGA

> Translation from *MeTHIC2* transcript sequence excluding intron 4

Met A S V Q A T S L S S A V C K N G S H K F P S G S F L P G F D G V L G R G S L K K E I W P R S L A T S V P R A T L T F D P P T T N S D K A K Q R K H
T V D P S S P D F L P L P S F E Q C F P R S T K E Y R E I V H E E S G H V L K V P F R R V H L S G D E P S F D N Y D T S G P Q N I S P R I G I P K L R K D
W V D R R E K L G A P R Y S Q **Met** Y Y A K Q G I I T E E **Met** L Y C A A R E K L D P E F V R S E V A R G R A I I P S N K K H L E L E P **Met** I V G R N F L
V K V N A N I G N S A V A S S I E E E V Y K V Q W A T **Met** W G A D T V **Met** D L S T G R H I H E T R E W I L R N S A V P V G T V P I Y Q A L E K V N
G I A E N L S W E V F R D T L I E Q A E Q G V D Y F T I H A G V L L R Y I P L T A K R **Met** T G I V S R G G S I H A K W C L A Y H K E N F A Y E H W D D
I L D I C N Q Y D V A L S I G D G L R P G S I Y D A N D T A Q F A E L L T Q G E L T R R A W E K D V Q V **Met** N E G P G H I P **Met** H K I P E N **Met** Q
K Q L E W C N E A P F Y T L G P L T T D I A P G Y D H I T S A I G A A N I G A L G T A L L C Y V T P K E H L G L P N R D D V K A G V I A Y K I S A H A A
D L A K G H P H A Q A W D D A L S K A R F E F R W **Met** D Q F A L S L D P **Met** T A **Met** S F H D E T L P S E G A K V A H F C S **Met** C G P K F C S
Met K I T E D V R K Y A E E H G Y G S A E E A V Q H G **Met** D A **Met** S A E F L A A K K T V S G E Q H G E V G G E I Y L P A S Y I K S S K R S **Met** **Stop**

> Translation from *MeTHIC2* transcript sequence including intron 4

Met A S V Q A T S L S S A V C K N G S H K F P S G S F L P G F D G V L G R G S L K K E I W P R S L A T S V P R A T L T F D P P T T N S D K A K Q R K H
T V D P S S P D F L P L P S F E Q C F P R S T K E Y R E I V H E E S G H V L K V P F R R V H L S G D E P S F D N Y D T S G P Q N I S P R I G I P K L R K D
W V D R R E K L G A P R Y S Q **Met** Y Y A K Q G I I T E E **Met** L Y C A A R E K L D P E F V R S E V A R G R A I I P S N K K H L E L E P **Met** I V G R N F L
V K V N A N I G N S A V A S S I E E E V Y K V Q W A T **Met** W G A D T V **Met** D L S T G R H I H E T R E W I L R N S A V P V G T V P I Y Q A L E K V N
G I A E N L S W E V F R D T L I E Q A E Q G V D Y F T I H A G V L L R Y I P L T A K R **Met** T G I V S R G G S I H A K W C L A Y H K E N F A Y E H W D D
I L D I C N Q Y D V A L S I G D G L R P G S I Y D A N D T A Q F A E L L T Q G E L T R R A W E K D V Q V **Met** N E G P G H I P **Met** H K I P E N **Met** Q
K Q L E W C N E A P F Y T L G P L T T D I A P G Y D H I T S A I G A A N I G A L G T A L L C Y V T P K E H L G L P N R D D V K A G V I A Y K I S A H A A
D L A K G H P H A Q A W D D A L S K A R F E F R W **Met** D Q F A L S L D P **Met** T A **Met** S F H D E T L P S E G A K V A H F C S **Met** C G P K F C S
Met K I T E D V R K Y A E E H G Y G S A E E A V Q H G **Met** D A **Met** S A E F L A A K K T V S G E Q H G E V G G E I Y L P A S Y I K S S K R **Stop** G S N
S N K P K K V F Y F G G K S R I A L S K **Stop** Y I N H L Q L Q V H C L L N H F T I **Stop** L A R L S K G S C S L L S **Stop** S L Q A K L I **Met** E D C L I L V S V
Met L L F E P **Stop** K **Stop** P C E I **Met** V T L C L I S H L C F L F G W T **Met** T F L T W N L Q F F L E F S C R S **Met** **Stop**

Supplementary Fig. S6. Translation of *MeTHIC1* and *MeTHIC2* transcript forms Ia (including intron 4) and Ib (excluding intron 4).

In the *MeTHIC1* **(A)** and *MeTHIC2* **(B)** transcript sequences, the five exons are highlighted in blue. Intron 4 is not highlighted. The corresponding protein sequences were obtained by translation of the transcripts excluding or including intron 4 using the ExPASy translate tool (Artimo *et al.*, 2012). In MeTHIC1 **(A)** and MeTHIC2 **(B)** protein sequences obtained from the transcripts, the open reading frames are highlighted in blue-green. For each *MeTHIC* homolog, the underlined amino acids correspond to sequence similarity between the two transcripts. The premature stop codon appearing in the transcript forms including intron 4 is highlighted in orange.

A

***AtTHIC* (At2g29630), 5'-UTR and 1000 bp upstream of the 5'-UTR**

TAGAGAGGGGATGGTTTTATGTACGGATCGGATCGTGCGGGGAAGACAAAATAGAAAAACAACGAGGGAGTTAGTTGCTTACATGTTG
TTTTTCAAAGATATTATTTTCTTCTTATTACATACACTTTTGAATTTGTTGATCGTGTTACTTACATAAAATTGCAGGTTAGGTCCCTTTGTTT
TCGCAGTTTTTGCAATTATTTCTCATATTTCTTAATATTGGGCTTTTCACATGTAATAAGCCCAACGATAAGACCATGACAATTTCTATACG
AAACATGATATAAATTCTTTGGATATACATTATGAATTTACGATATACAATTAGTTTGTTTAAATATCAAAATATAAATGCGTCAATGGTTG
TTGTTACTTGTGAGATTATCTTTCTATTTAAGAAGAATAATTCTCTTCGTAGATAAATTTTTAAAATAATTTTCCGAGTTTTCTAATGTTTCT
AGATATGATTTGATTTGAACAATTAATTCGTGGTTCTTTGAATGAATATATCGACTGTATTTGATTTCAGTTAAACTGATAATAATTGTCAT
TTACGTCTCAAAAGAATTGAAATATCATGTCTCTCAAGATATGGACTTACATATTGTTATGCATTATTTATCAAAATATGTGGACAAAACAT
AATATCAATGTCGCTTTCAGAATAATTGAACAACAGATATTGAGAAATCAATTTTTATGGTTATATCAATTGTCATTGCCAACATCTATTAC
ATAGTAACAGTCCAATTTACATTACAATGGTAATTCAATGAAGGTAATTTACTTTTTATTGGTTTACTCGTGAAACGACGTTCTCCTCCTCA
CGTACCTTATCTTAATATCCTGATCAACGGACACCAATTTTCGACAAAATATCTGAGAAAGAGGACACGTCAGCAAGCCTTTCGCTTTAG
GCTGCATTGGGCCGTGACAATATTCAGACGATTCAGGAGGTTCGTTCCTTTTTTAAAGGACCCTAATCACTCTGAGTACCACTGACTCACT
CAGTGTGCGCGATTCATTTCAAAAACGAGCCAGCCTCTTCTTCCTTCGTCTACTAGATCAGATCCAAAGCTTCCTCTTCCAGCTATG

B

***MeTHIC1* (Manes.02G121700), 5'-UTR and 1000 bp upstream of the 5'-UTR**

TATCTATAAACGTATGGTGATGGTATGAAGGGTGAACACTGAACAGGCAGCCCGTTTCTTAGTTTGCCCTTTTTTCCTCCTCGCCAATCGA
GCTATTGACGGTGGAGCATTGGAGCTATCATTATCTTCATTAATCCAATCTTTAATGATCTTGAGTTATAACAACTCCGTTTGATCGGGTCA
ACGCTTTGCATAAATTTTGTTGCCTGGTTACATTGTGTCTAATTTAGGAAATGTTTTTTAAATTCAGAACCTCATGATGAAAATTTATTACA
ATTACGTTTTTCTCGAAAGTTTCTATAGTAATTTTATGTGAATTAAGAAAAGGAAATTGATATAGTTAAATCAATAAATTATATTATTTTTCT
TGATGCATTGTTTTTGTCTGAATTTTTCTTAATGAATTCCCTATTCGTAAATTTACAATTTAGTTTATACTGTTTAATGATAAATTATTTATTC
TATGAATTCTCATTTTACTAGCAAAATATTCCTATTATTAAAATTTATTATTAAATAACATCCTAATCATTTGAATTTTAATTTTTTTATAATA
ATTTAATCATTATAGTTTTTAATATTTACAATAATTTAGCATTTACAGTTCTACGCTTTGAAACAATTTATTTCCATCGATTTAGATTAATTTT
TTCAATTTATATTAATGGTGAATTTAATAGAAGGATTATTTAATCAATAAAATAAAAACTTAAAAATATTTTAAAAAAAAATAAGAATTAAG
TGGTAGATTTTATTAAATTTTAATGAATATTAGTGTAGTTTATTTTAAATTTTACTTGTGAGTTCGAATATAAAATGATTTTAGACCAAAAG
AAAACATTAATATATTCTCCCAATTTTAATAACCCTATCCTATCCTCCTACTATCCAGTATCCACGTAAGCATTCACTTTTGATAACTTTTTCT
ACTTTCAAGTCCCTATAAATGAAAAGCCAATCAGAGCTCCTGACATCGCCTACCTTTGGCGCCTACCTTTGGCTGCGTTCAGCGGTTCAGT
TTTATGTACTCACTCTCTCTTCTCGAGAGATAAGACTCTTCTCCTGCAATTCCCCTTTCCTATCTCTCTGGTGAACGAAATG

C

***MeTHIC2* (Manes.01G164200), 5'-UTR and 1000 bp upstream of the 5'-UTR**

AATTAATTAACCAAACAAATAAATTGAAATCAAGAATCAAAATGAGAGTCTATCAATATAATTAAAAATGTATTTAGAACTTGAGGGGTT
AATATCTCCTAATTTAAATTCATGTAACAGTCTTTGCCTTTCTTGTCTCAAAATTAATAAAAATTACTAAATTCTTTATCAATTTTATTAAGCT
ATTTTTTCAACACACAAAAATAAATAATAATCAAATTAGCAAATATACAAATATCTTTTTTTATTTTATTTTACTATATAAATAATATAGTAG
GATGTGAATTTAATAATATCAAATAATTCAGATATTTATAATAGAGAGGCCATAATTAAATTTTATTAGTTACTATGAAAACTTCATTATAT
TCAAATAAATAATAATTATCTGTTATTATTTTAATATTATGATAATTATTTTTAATATTCTTAAATAGTATTTAAATTTATTATTTTANNNNN
NNNNNNNNNNNNNNNNNNNNNNNNNNNNNNNNNNNNNNNNNNNNNNNNNNNNNNNNNNNNNNNNNNNNNNNNNNNNNNNNNNNNNNN
NNNNNNNNNNNNNNNNNNNNNNNNNNNNNNNNNNNNNNNNNNNNNNNNNNNNNNNNNNNNNNNNNNNNNNNNNNNNNNNNNNNNNNN
NNNNNNNNNNNNNNNNNNNNNNNNNNNNNNNNNNNNNNNNNNNNNNNNNNNNNNNNNNNNNNTTTAAATAATTAAATATAAATATAAATCA
TTTAAATATTTTTCTCATTTTTTTCTTTCCTTCTACTTTTGTTTATATATAATATAGATTAAAAAAAAAGGGTTATAGGCGCCGCCACCGGGT
GAATCTGCGAAATGATCTAACGGTCCTCCATTGAGGAGTGTACCTCGAGATGTGCAGACGAGGGTAACCTGCACCCCTGTAGATTTTTAC
AGATATATCTGACCTCCCCACGTAAGCATTCGATTTTGGCGTTTGAGTGGTTGCAGGTGTAGCTGTACCTGCCATATATAAATACAAAGG
CAGAGCGAAGCTTTAAGCTCCCGTGACCTCGCTTACCTTGGGCTGCGTTCCGCTGTTCAGTTCTTATCTCCTCCCTCACAGTCATCAAGATA
TAAAGACTCCTCGCTGCATTTCCTCGTTTTTCTCTCTTTTGTGACGAAATG

Supplementary Fig. S7. Analysis for a CCA1 binding motif upstream of the 5'-UTR of Arabidopsis and cassava *THIC* genes.

The CCA1 binding motif (AAAATATCT) has been reported to be located 128 bp upstream of the 5'-UTR of *AtTHIC* (Bocobza *et al.*, 2013) **(A)**, and could not be detected in the 5'-UTR of either *MeTHIC1* **(B)** or *MeTHIC2* **(C)**. The 5'-UTR sequence is highlighted in blue-green, the start codon (ATG) is highlighted in blue and the CCA1 binding motif is underlined in vermilion. Sequences predicted in Phytozome, *Arabidopsis thaliana* TAIR10 (Lamesch *et al.*, 2012) and *Manihot esculenta* v6.1 (Prochnik et al., 2012) genomes.

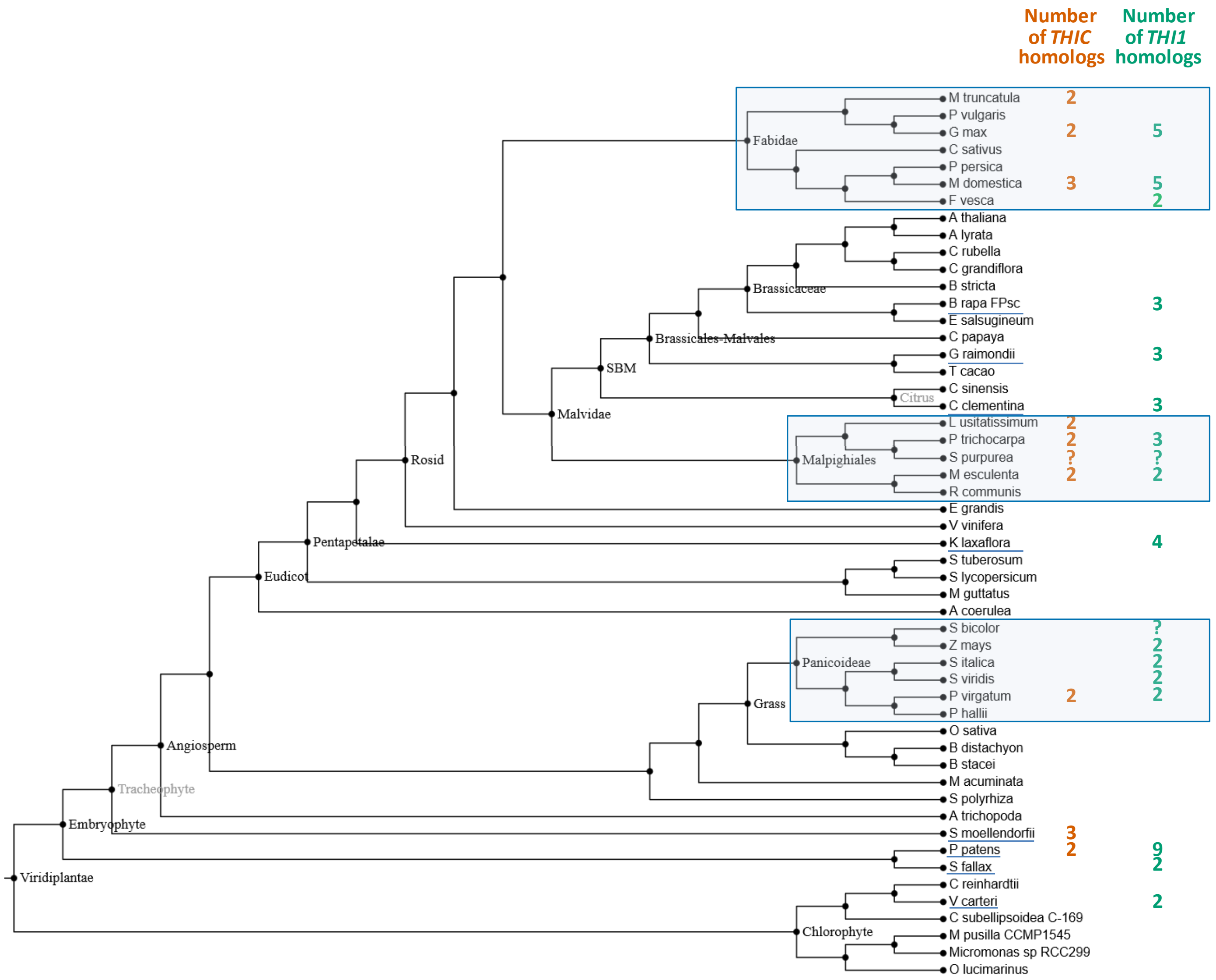

Supplementary Fig. S8. Number of *THIC* and *THI1* homologs in the genomes available in the Phytozome database.
Phylogenetic tree representation of the species available in the Phytozome database (Goodstein *et al.*, 2012). In vermilion and blue-green are the number of *THIC* and *THI1* homologs annotated in Phytozome for different plant species, showing a duplication of one or both vitamin B_1 biosynthesis *de novo* genes. Plant species with multiple *THIC* and/or *THI1* homologs are grouped in three main clades (Fabidae, Malpighiales and Panicoideae), which are highlighted in blue boxes.

```
AtTHIC      MAASVHCTLMSVVCNNKNHSARPKLPNSSLLPGFDVVVQAAATRF-----KKETTTTRAT
MeTHIC1     MASVQATSFASSVCKN----GTQKFPSSSFLPGFDVVAGRGFIKKETLPRSLISSIPRAT
MeTHIC2     MASVQATSLSSAVCKN----GSHKFPSGSFLPGFDGVLGRGSLKKEIWPRSLATSVPRAT
            **:    :: * **:*    .  *:*..*:***** *   .  :      .  ::  ***

AtTHIC      LTFDPPTTNSERAKQRKHTIDPSSPDFQPIPSFEECFPKSTKEHKEVVHEESGHVLKVPF
MeTHIC1     LTFDPPTTNSDKIKQRKHTLDPSSPDFLPLPSFEQCFPRSTKEYREVVHEQTGHVLKVPF
MeTHIC2     LTFDPPTTNSDKAKQRKHTVDPSSPDFLPLPSFEQCFPRSTKEYREIVHEESGHVLKVPF
            **********:: ******:******* *:****:***:****::*:***::********

AtTHIC      RRVHLSGGEPAFDNYDTSGPQNVNAHIGLAKLRKEWIDRREKLGTPRYTQMYYAKQGIIT
MeTHIC1     RRVHLSGDEPCFDNYDTSGPQNISPRTGLPKLRKDWVDRREKLGTPRYTQMYYAKQGIIT
MeTHIC2     RRVHLSGDEPSFDNYDTSGPQNISPRIGIPKLRKDWVDRREKLGAPRYSQMYYAKQGIIT
            ******* **.***********:. : *: ****:*:*******:***:***********

AtTHIC      EEMLYCATREKLDPEFVRSEVARGRAIIPSNKKHLELEPMIVGRKFLVKVNANIGNSAVA
MeTHIC1     EEMLYCAVREKLDPEFVRSEVARGRAIIPSNKKHLELQPMIVGRNFLVKVNANIGNSAVA
MeTHIC2     EEMLYCAAREKLDPEFVRSEVARGRAIIPSNKKHLELEPMIVGRNFLVKVNANIGNSAVA
            *******.*****************************:******:***************

AtTHIC      SSIEEEVYKVQWATMWGADTIMDLSTGRHIHETREWILRNSAVPVGTVPIYQALEKVDGI
MeTHIC1     SSIEEEVYKVQWATMWGADTVMDLSTGRHIHETREWILRNSAVPVGTVPIYQALEKVNGI
MeTHIC2     SSIEEEVYKVQWATMWGADTVMDLSTGRHIHETREWILRNSAVPVGTVPIYQALEKVNGI
            *******************:*************************************:**

AtTHIC      AENLNWEVFRETLIEQAEQGVDYFTIHAGVLLRYIPLTAKRLTGIVSRGGSIHAKWCLAY
MeTHIC1     AENLSWEVFRETLIEQAEQGVDYFTIHAGVLLRYIPLTAKRMTGIVSRGGSIHAKWCLAY
MeTHIC2     AENLSWEVFRDTLIEQAEQGVDYFTIHAGVLLRYIPLTAKRMTGIVSRGGSIHAKWCLAY
            ****.*****:******************************:******************

AtTHIC      HKENFAYEHWDDILDICNQYDVALSIGDGLRPGSIYDANDTAQFAELLTQGELTRRAWEK
MeTHIC1     HKENFAYEHWDDILDICNQYDVALSIGDGLRPGSIYDANDTAQFAELLTQGELTRRAWEK
MeTHIC2     HKENFAYEHWDDILDICNQYDVALSIGDGLRPGSIYDANDTAQFAELLTQGELTRRAWEK
            ************************************************************

AtTHIC      DVQVMNEGPGHVPMHKIPENMQKQLEWCNEAPFYTLGPLTTDIAPGYDHITSAIGAANIG
MeTHIC1     DVQVMNEGPGHIPMHKIPENMQKQLEWCNEAPFYTLGPLTTDIAPGYDHITSAIGAANIG
MeTHIC2     DVQVMNEGPGHIPMHKIPENMQKQLEWCNEAPFYTLGPLTTDIAPGYDHITSAIGAANIG
            ***********:************************************************

AtTHIC      ALGTALLCYVTPKEHLGLPNRDDVKAGVIAYKIAAHAADLAKQHPHAQAWDDALSKARFE
MeTHIC1     ALGTALLCYVTPKEHLGLPNRDDVKAGVIAYKISAHAADLAKGHPHAQAWDDALSKARFE
MeTHIC2     ALGTALLCYVTPKEHLGLPNRDDVKAGVIAYKISAHAADLAKGHPHAQAWDDALSKARFE
            *********************************:******** *****************

AtTHIC      FRWMDQFALSLDPMTAMSFHDETLPADGAKVAHFCSMCGPKFCSMKITEDIRKYAEENGY
MeTHIC1     FRWMDQFALSLDPMTAMSFHDETLPSEGAKVAHFCSMCGPKFCSMKITEDVRKYAEEHGY
MeTHIC2     FRWMDQFALSLDPMTAMSFHDETLPSEGAKVAHFCSMCGPKFCSMKITEDVRKYAEEHGY
            *************************::***********************:******.**

AtTHIC      GSAEEAIRQGMDAMSEEFNIAKKTISGEQHGEVGGEIYLPESYVKAAQK*--
MeTHIC1     GSAEEAVQHGMDAMSAEFLAAKKTVSGEQHGEVGGEIYLPESYIRSSERSI*
MeTHIC2     GSAEEAVQHGMDAMSAEFLAAKKTVSGEQHGEVGGEIYLPASYIKSSKRSM*
            ******:::****** **  ****:*************** **::::::
```

Supplementary Fig. S9. Alignment of THIC protein sequences from Arabidopsis and cassava

Alignment of AtTHIC (encoded by At2g29630), MeTHIC1 (encoded by Manes.02G121700, 87% identity with AtTHI1) and MeTHIC2 (encoded by Manes.01G164200, 87% identity with AtTHI1) protein sequences. The residues involved in the main binding sites are conserved in MeTHIC1 and MeTHIC2. The residues responsible for the iron sulfur cluster (consensus amino acid motif CX_2CX_4C, where X represents any amino acid) are highlighted in purple; the residues responsible for the AIR substrate binding site are highlighted in yellow; the conserved histidine residues responsible for the iron binding site are highlighted in blue. The sequence in blue box corresponds to the central domain of the protein (residues 224 - 519) containing the $(\beta/\alpha)_8$ TIM barrel, where the active site is located. The differences in amino acid sequence between MeTHIC1 and MeTHIC2 in the central domain are depicted in vermilion.

Supplementary Table S1. Description of the 41 cassava accessions selected for quantification of vitamin B_1 content.

	Accession number	Synonyms	Common name	Species	Biological status	Origin
Africa	TMe-3	-	2nd Agric	*Manihot esculenta*	Traditional accession/Landrace	Nigeria
	TMe-7	Tme-3349, TMe-3350, TMe-3321, TMe-3352, TMe-3440	Oko-iyawo	*M. esculenta*	Traditional accession/Landrace	Nigeria
	TMe-14	-	Abbey-ife	*M. esculenta*	Traditional accession/Landrace	Nigeria
	TMS 30001	-	MNga-1	*M. esculenta*	Improved-Line (lost pedigree)	Nigeria
	TMS 30555	-	-	*M. esculenta*	Improved-Line (58308 x Oyarugba Dudu)	Nigeria
	TMS 30572	-	-	*M. esculenta*	Improved-Line (58308 x Branca de Santa Caterina)	Nigeria
	cv. 60444	TMS 60444	-	*M. esculenta*	Benchmark model accession	Nigeria
	Ebwanateraka	-	-	*M. esculenta*	Traditional accession/Landrace	Uganda
	Kibandameno WT64	-	-	*M. esculenta*	Traditional accession/Landrace	Kenya
	Kibaha	-	-	*M. esculenta*	Improved variety	Tanzania
	KBH 2006/18	-	-	*M. esculenta*	Elite breeding lines from the IITA	Tanzania
	KBH 2006/26	-	-	*M. esculenta*	Elite breeding lines from the IITA	Tanzania
	T 200	-	-	*M. esculenta*	Traditional accession/Landrace	South Africa
Asia	KU50	-	Kasetsart 50	*M. esculenta*	Improved-Line	Thailand
	SC8	-	-	*M. esculenta*	Traditional accession/Landrace	China
	SC124	-	-	*M. esculenta*	Traditional accession/Landrace	China
	TAI 8	-	Cmr 246343(Rayong 60)	*M. esculenta*	Improved-Line	Thailand
	TAI 3	-	Rayong 3	*M. esculenta*	Improved-Line	Thailand
	H226	-	-	*M. esculenta*	Improved commercial hybrid	India
	KM140	-	-	*M. esculenta*	Improved line (KM98-1 x KM 36)	Vietnam
	KM397	-	-	*M. esculenta*	Improved line (SM937-26 SM937-26 x) x (x BKA900 BKA900)	Vietnam
	11SA12	-	-	*M. esculenta*	-	Vietnam

Supplementary Table S1 (continued)

	Accession number	Synonyms	Common name	Species	Biological status	Origin
South and Central America	ECU 72	072	Injerta	*M. esculenta*	Traditional accession/Landrace	Ecuador
	COL 22	COR-313	Uvita	*M. esculenta*	Traditional accession/Landrace	Colombia
	PER 183	-	Eeat 1	*M. esculenta*	Traditional accession/Landrace	Peru
	COL 1505	BRA-001007, CMC 76	Ucv 2096	*M. esculenta*	Traditional accession/Landrace	Venezuela
	CM 2177-2	-	Cebucan	*M. esculenta*	Improved-Line (CM 430-37 x CM 840-138)	Colombia
	MEX 95	-	-	*M. esculenta*	Traditional accession/Landrace	Mexico
	BRA 132	BGM 0186, BRA-006670	Mendubi	*M. esculenta*	Traditional accession/Landrace	Brazil
	ARG 13	-	B1-15-3-73	*M. esculenta*	Traditional accession/Landrace	Argentina
	GUA 79	884	Yuca Colorada	*M. esculenta*	Traditional accession/Landrace	Guatemala
	CM 523-7	-	Catumare	*M. esculenta*	Improved-Line (COL 655A x COL 1515)	Colombia
	SG 107-35	-	-	*M. esculenta*	Improved-Line (VAR 5)	Colombia
	PSE XXX-1	-	-	*M. pseudoglaziovii*	Wild	Brazil
	TST XXX-18	-	-	*M. tristis*	Wild	Brazil
	VEN 25	UCV 2076, 025	Querepa Amarga	*M. esculenta*	Traditional accession/Landrace	Venezuela
	BRA 685	BRA-068471	Amarelinha	*M. esculenta*	Traditional accession/Landrace	Brazil
	CM 6438-14	-	-	*M. esculenta*	Improved-Line (CM 1335-4 x CM 2298-3)	Colombia
	BRA 293	BGM 0549, BRA-012611	Amansa Burro	*M. esculenta*	Traditional accession/Landrace	Brazil
	BRA 325	BGM 1165, BRA-002291	Tapioqueira	*M. esculenta*	Traditional accession/Landrace	Brazil
	BRA 222	BGM 0374, BRA-002356	Verdinha	*M. esculenta*	Traditional accession/Landrace	Brazil

Supplementary Table S2. Primers used for PCR amplification of *MeTHIC1* and *MeTHIC2* 3'-UTR splice variants.

Target gene	Gene reference	Primer name	Primer sequence (5' - 3')
MeTHIC1	Manes.02G121700	F_{THIC1}<br>R_{THIC1}	CCGGAAAGTTACATTAGATCTTCTGAA<br>GCAAGCAATTAAAAGGATTAAC
MeTHIC2	Manes.01G164200	F_{THIC2}<br>R_{THIC2}	GCCAGCAAGTTATATTAAATCCTCTAAG<br>CACTTGTTATATCGGATATACG
**-**	-	3'-RACE primer	GCTGTCAACGATACGCTACGTAACG
**-**	-	3'-RACE nested	CGCTACGTAACGGCATGACAGTG

Supplementary Table S3. Primers used for RT-qPCR analysis.
It should be noted that primer pairs MeTHIC1-F/MeTHIC1-R and MeTHIC2-F/MeTHIC2-R can amplify *THIC* transcripts Ib, II and III but not Ia.

Target gene	Gene reference	Primer name	Primer sequence (5' - 3')
MePP2A	Manes.09G039900	PP2A-F<br>PP2A-R	TGCAAGGCTCACACTTTCATC<br>CTGAGCGTAAAGCAGGGAAG
MeUBQ10	Manes.07G019300	UBQ10_F<br>UBQ10-R	TGCATCTCGTTCTCCGATTG<br>GCGAAGATCAGTCGTTGTTGG
MevATPs	Manes.08G064200	vATPs_F<br>vATPs_R	GTTGAATGGCTTTGTGCTCAG<br>CCATCTGCGTGAACAAAAGAA
MeTHIC1	Manes.02G121700	MeTHIC1_F<br>MeTHIC1-R	ATGAGTGCTGAGTTCCTGGCTG<br>GCTTTTTTGACCAAATCCCTGG
MeTHIC2	Manes.01G164200	MeTHIC2-F<br>MeTHIC2-R	GTTAGTGGAGAACAACATGGTGAAG<br>CATATCCATCATCCTCACAACTCAGA
MeTHI1a	Manes.15G075600	MeTHI1a-F<br>MeTHI1a-R	CATGGAGGTTGCGGAGATTG<br>AAACTCTGGGCGAAGGGTAA
MeTHI1b	Manes.03G123800	MeTHI1b-F<br>MeTHI1b-R	CATGGAAGTTGCAGAGATTG<br>CAAATTCTGGGCTTCGCTTGT

Supplementary Table S4. Phenotypic characterization of greenhouse grown cassava accessions.

(A) Evaluation of plant height, above-ground (leaves and stem) fresh weight and storage root fresh weight in 41 cassava accessions. Average ± S.D. of 3 biological replicates (except for TMS 30572 (n=2) for aboveground phenotype and for TMS 30572 (n=2), MEX 95 (n=2) and PSE XXX-1 (n=2) for under-ground phenotype). Tukey's multiple comparison test ($p < 0.05$). **(B)** Repeated independent evaluation of plant height, above-ground fresh weight, number of leaves, leaf retention (leaves retained at harvest time/total number of leaves), under-ground fresh weight and number of storage roots in 18 selected accessions. Average ± S.D. of 4 biological replicates (except for CM2177-2 (n=1) and TST XXX-18 (n=3) for above-ground phenotype and CM2177-2 (n=1), TST XXX-18 (n=1) and MEX95 (n=3) for underground phenotype). Tukey's multiple comparison test ($p < 0.05$).

	**A. Preliminary phenotypic characterization of 41 cassava accessions**			**B. Repeated independent phenotypic characterization of 18 selected cassava accessions**					
**Accessions**	**Plant height (cm)**	**Above-ground FW (g)**	**Under-ground FW (g)**	**Plant height (cm)**	**Above-ground FW (g)**	**Number of leaves**	**Leaf retention (%)**	**Under-ground FW (g)**	**Number of storage roots**
TMe-3	**113.3** [±18.7][abcdef]	**51.0** [±9.5][abcdef]	**47.2** [±16.2][abc]	**90.6** [±20.6][abcd]	**55.7** [±12.6][bcd]	**20.3** [±2.2][abc]	**84.3** [±7.3][abcd]	**26.7** [±5.1][ab]	**3.0** [±1.6][a]
TMe-7	**102.0** [±13.2][abcdef]	**64.5** [±4.7][bcdefghi]	**39.7** [±14.1][abc]	**112.0** [±23.1][cde]	**79.9** [±10.0][cdef]	**24.3** [±1.0][cde]	**98.0** [±2.3][e]	**38.4** [±3.3][abc]	**4.8** [±0.5][abc]
TMe-14	**122.0** [±26.2][bcdef]	**90.0** [±19.4][hij]	**35.0** [±16.4][abc]						
TMS 30001	**84.3** [±11.8][abcde]	**45.7** [±4.7][abcd]	**35.8** [±11.7][abc]						
TMS 30555	**112.3** [±18.6][abcdef]	**73.6** [±13.6][bcdefghij]	**37.3** [±23.1][abc]						
TMS 30572	**97.0** [±12.7][abcdef]	**43.3** [±7.2][abcd]	**53.4** [±6.4][abc]	**90.0** [±22.6][abcd]	**61.3** [±17.6][bcde]	**21.5** [±2.6][cd]	**95.6** [±5.9][de]	**33.1** [±7.8][abc]	**8.5** [±1.0][cd]
cv. 60444	**94.0** [±21.0][abcdef]	**63.8** [±10.8][bcdefghi]	**61.7** [±13.3][abc]	**82.1** [±19.6][abcd]	**58.6** [±15.5][bcde]	**22.5** [±1.7][cde]	**97.0** [±3.7][e]	**36.6** [±1.5][abc]	**7.0** [±1.4][abcd]
Ebwanateraka	**135.0** [±26.9][cdefg]	**81.9** [±17.5][efghij]	**60.0** [±20.3][abc]						
Kibandameno	**122.0** [±15.7][bcdef]	**64.8** [±7.6][bcdefghi]	**32.7** [±18.2][abc]						
Kibaha	**129.0** [±9.5][cdefg]	**88.7** [±12.5][ghij]	**55.5** [±9.2][abc]	**125.4** [±3.5][de]	**68.0** [±5.8][bcdef]	**24.3** [±1.0][cde]	**91.7** [±5.6][bcde]	**19.9** [±8.5][a]	**4.5** [±1.3][ab]
KBH 2006/18	**97.7** [±25.3][abcdef]	**45.2** [±9.3][abc]	**79.3** [±20.1][c]	**103.3** [±30.2][cd]	**58.6** [±9.4][bcde]	**25.0** [±0.8][de]	**96.3** [±2.9][de]	**51.8** [±17.7][bc]	**5.3** [±0.5][abcd]
KBH 2006/26	**159.7** [±14.3][fg]	**77.7** [±5.9][cdefghij]	**41.5** [±12.4][abc]						
T 200	**96.0** [±15.6][abcdef]	**51.1** [±6.2][abcdef]	**0**						
ECU 72	**107.3** [±33.3][abcdef]	**65.7** [±8.7][bcdefghi]	**51.8** [±26.5][abc]						
COL 22	**134.3** [±26.7][cdefg]	**72.3** [±11.6][bcdefghij]	**43.6** [±12.5][abc]	**127.1** [±15.1][de]	**82.2** [±6.3][def]	**23.8** [±1.5][cde]	**94.0** [±5.2][de]	**35.9** [±13.0][abc]	**7.8** [±2.1][bcd]
PER 183	**105.0** [±18.7][abcdef]	**48.3** [±9.2][abcde]	**32.0** [±5.5][abc]						
COL 1505	**96.0** [±25.5][abcdef]	**58.0** [±16.8][bcdefghi]	**35.2** [±19.0][abc]	**93.8** [±14.6][bcd]	**61.4** [±11.6][bcde]	**21.5** [±1.9][cd]	**87.1** [±9.0][bcde]	**20.7** [±4.6][a]	**3.8** [±1.0][a]
CM 2177-2	**92.7** [±10.1][abcdef]	**51.1** [±5.1][abcdef]	**29.8** [±13.2][ab]	**85.0**	**67.4**	**25.0**	**96.2**	**16.7**	**2.0**
MEX 95	**102.0** [±7.0][abcdef]	**59.2** [±8.9][bcdefghi]	**26.8** [±18.9][abc]	**107.8** [±29.9][cd]	**66.7** [±8.2][bcdef]	**23.0** [±2.4][cde]	**81.3** [±5.7][ab]	**8.1** [±3.5][a]	**4.0** [±0.0][ab]
BRA 132	**71.3** [±17.9][abc]	**54.7** [±10.7][abcdefg]	**48.0** [±15.8][abc]	**64.5** [±2.7][abc]	**61.6** [±15.0][bcde]	**23.5** [±3.1][cde]	**97.2** [±3.7][e]	**53.1** [±32.4][bc]	**5.5** [±1.9][abcd]

Supplementary Table S4 (continued)

	A. Preliminary phenotypic characterization of 41 cassava accessions			B. Repeated independent phenotypic characterization of 18 selected cassava accessions					
**Accessions**	**Plant height (cm)**	**Above-ground FW (g)**	**Under-ground FW (g)**	**Plant height (cm)**	**Above-ground FW (g)**	**Number of leaves**	**Leaf retention (%)**	**Under-ground FW (g)**	**Number of storage roots**
ARG 13	**141.7** [±15.3]^{defg}	**76.6** [±7.4]^{bcdefghi}	**50.9** [±10.6]^{abc}	**157.9** [±6.0]^{e}	**88.2** [±4.9]^{ef}	**21.0** [±0.0]^{bcd}	**73.7** [±1.5]^{a}	**27.1** [±6.8]^{ab}	**5.5** [±1.3]^{abcd}
GUA 79	**112.7** [±16.3]^{abcdef}	**72.5** [±16.0]^{bcdefghij}	**20.5** [±10.3]^{a}	**158.4** [±6.2]^{e}	**96.5** [±4.5]^{f}	**20.5** [±1.0]^{abcd}	**97.7** [±4.5]^{e}	**10.0** [±0.9]^{a}	**3.3** [±1.7]^{a}
CM 523-7	**141.0** [±7.9]^{defg}	**79.5** [±6.8]^{defghij}	**33.1** [±12.3]^{abc}						
SG 107-35	**130.0** [±9.6]^{cdefg}	**76.4** [±5.2]^{bcdefghij}	**22.8** [±7.0]^{a}						
PSE XXX-1	**138.0** [±18.2]^{cdefg}	**51.6** [±9.6]^{abcdef}	**18.4** [±6.6]^{a}	**98.3** [±18.9]^{bcd}	**41.0** [±12.4]^{ab}	**16.0** [±0.8]^{a}	**82.2** [±4.5]^{abc}	**11.2** [±4.5]^{a}	**5.3** [±1.7]^{abcd}
TST XXX-18	**138.7** [±12.4]^{cdefg}	**71.1** [±5.9]^{bcdefghij}	**26.4** [±7.8]^{ab}	**111.7** [±37.6]^{cde}	**41.7** [±19.0]^{ab}	**22.3** [±3.1]^{cde}	**98.7** [±2.2]^{e}	**32.13**	**6.0**
VEN 25	**151.3** [±3.5]^{efg}	**63.8** [±3.4]^{bcdefghi}	**29.3** [±6.2]^{ab}						
BRA 685	**139.3** [±8.3]^{cdefg}	**90.8** [±0.9]^{ij}	**30.1** [±12.1]^{ab}						
CM 6438-14	**137.3** [±4.2]^{cdefg}	**72.6** [±8.8]^{bcdefghij}	**33.6** [±17.1]^{abc}						
BRA 293	**149.7** [±31.3]^{efg}	**83.4** [±12.4]^{fghij}	**21.3** [±5.5]^{a}						
BRA 325	**195.0** [±12.2]^{g}	**89.4** [±7.4]^{hij}	**46.6** [±6.4]^{abc}						
BRA 222	**100.3** [±21.1]^{abcdef}	**56.6** [±6.0]^{bcdefgh}	**32.3** [±3.0]^{abc}						
KU50	**122.3** [±5.1]^{bcdef}	**65.7** [±5.0]^{bcdefghi}	**55.7** [±7.0]^{abc}	**114.8** [±16.5]^{de}	**74.7** [±13.5]^{cdef}	**22.3** [±1.0]^{cd}	**93.9** [±4.1]^{cde}	**34.2** [±6.3]^{abc}	**4.3** [±1.3]^{ab}
SC8	**47.0** [±6.2]^{a}	**43.0** [±7.3]^{ab}	**58.2** [±7.7]^{abc}	**50.5** [±16.9]^{ab}	**51.3** [±16.1]^{abc}	**27.0** [±1.4]^{e}	**94.8** [±1.8]^{cde}	**24.2** [±12.0]^{ab}	**5.8** [±2.5]^{abcd}
SC124	**92.3** [±67.2]^{abcde}	**51.2** [±12.9]^{abcdef}	**32.0** [±21.3]^{abc}						
TAI 8	**75.7** [±27.5]^{abcd}	**58.6** [±14.4]^{bcdefghi}	**63.5** [±17.4]^{abc}						
TAI 3	**55.3** [±11.2]^{ab}	**21.4** [±4.6]^{a}	**66.2** [±30.7]^{abc}	**43.0** [±7.1]^{a}	**25.4** [±4.2]^{a}	**16.5** [±1.7]^{ab}	**84.6** [±4.0]^{abcd}	**59.3** [±11.0]^{c}	**9.0** [±2.4]^{d}
H226	**117.7** [±28.9]^{abcdef}	**100.7** [±21.5]^{j}	**40.1** [±15.7]^{abc}						
KM140	**102.0** [±17.6]^{abcdef}	**57.5** [±9.0]^{bcdefghi}	**72.8** [±9.4]^{bc}						
KM397	**120.0** [±20.0]^{bcdef}	**46.1** [±3.2]^{abcd}	**19.1** [±4.0]^{a}						
11SA12	**118.7** [±11.8]^{bcdef}	**67.7** [±9.8]^{bcdefghij}	**44.2** [±20.0]^{abc}						

Supplementary Table S5. Preliminary quantification of vitamin B_1 in 41 cassava accessions using a yeast bioassay.

Leaf **(A)** and storage root **(B)** vitamin B_1 contents in greenhouse grown cassava accessions. Accessions with a vitamin B_1 content below the 25th percentile of the distribution were considered as accessions with low vitamin B_1 content, whereas those with a vitamin content above the 75th percentile were considered as accessions with high vitamin B_1 content. Low, intermediate and high vitamin B_1 accessions selected for a repeated independent quantification are in blue-green, blue and vermilion, respectively. Average ± S.D. of 3 biological replicates except TMS 30572 (n=2) for leaves and TMS 30572 (n=2), MEX 95 (n=2), PSE XXX-1 (n=2) for storage roots. T200 did not produce any storage roots. Tukey's multiple comparison test ($p < 0.05$). Maximum fold difference (highest/lowest vitamin B_1 content) and S.D. calculated with Taylor series linearization method.

A LEAVES

Accession	Vitamin B_1 (ng mg FW^{-1})	
BRA 132	**1.78** [±0.63][a]	Below the 25th percentile of vitamin B_1 content distribution
COL 22	**2.24** [±0.39][a]	
BRA 222	**2.46** [±0.80][a]	
TMS 30572	**2.50** [±0.99][ab]	
TST XXX-18	**2.64** [±0.65][ab]	
TAI 3	**2.66** [±0.68][ab]	
TAI 8	**2.68** [±0.57][ab]	
KU50	**2.71** [±0.67][ab]	
BRA 685	**3.29** [±1.02][ab]	Between the 25th and 75th percentile of vitamin B_1 content distribution
MEX 95	**3.31** [±0.93][ab]	
VEN 25	**3.48** [±1.00][ab]	
SC124	**3.49** [±1.37][ab]	
KM397	**3.55** [±0.96][ab]	
KM140	**3.64** [±0.59][ab]	
KBH 2006/18	**3.64** [±1.14][ab]	
CM 6438-14	**3.69** [±1.07][ab]	
BRA 325	**3.70** [±0.44][ab]	
TMS 30001	**3.82** [±0.34][ab]	
PSE XXX-1	**3.84** [±2.38][ab]	
KBH 2006/26	**3.85** [±1.52][ab]	
H226	**3.99** [±0.26][ab]	
BRA 293	**4.05** [±2.10][ab]	
T 200	**4.14** [±0.29][ab]	
SC8	**4.26** [±1.70][ab]	
SG 107-35	**4.43** [±1.11][ab]	
TMS 30555	**4.44** [±0.74][ab]	
CM 2177-2	**4.50** [±1.34][ab]	
Kibandameno	**4.51** [±1.14][ab]	
Ebwanateraka	**4.57** [±1.10][ab]	
PER 183	**4.84** [±0.48][ab]	
COL 1505	**4.95** [±1.60][ab]	Above the 75th percentile of vitamin B_1 content distribution
TMe-3	**5.08** [±2.05][ab]	
TMe-7	**5.17** [±1.54][ab]	
TMe-14	**5.18** [±0.86][ab]	
11SA12	**5.23** [±3.35][ab]	
GUA 79	**5.25** [±3.28][ab]	
cv. 60444	**5.30** [±1.20][ab]	
Kibaha	**5.45** [±2.46][ab]	
CM 523-7	**5.98** [±1.98][ab]	
ECU 72	**6.14** [±3.87][ab]	
ARG 13	**7.52** [±0.27][b]	
Maximum fold difference		**4.22** [±0.37]

B STORAGE ROOTS

Accession	Vitamin B_1 (ng mg FW^{-1})	
CM 2177-2	**1.01** [±0.12][a]	Below the 25th percentile of vitamin B_1 content distribution
KBH 2006/18	**1.24** [±0.19][ab]	
TST XXX-18	**1.41** [±0.63][abc]	
CM 6438-14	**1.49** [±0.47][abc]	
SC124	**1.56** [±0.29][abc]	
BRA 325	**1.73** [±0.29][abcd]	
KBH 2006/26	**1.75** [±1.11][abcd]	
KU 50	**1.76** [±0.48][abcd]	
BRA 222	**1.79** [±0.29][abcd]	
MEX 95	**1.81** [±0.42][abcd]	Between the 25th and 75th percentile of vitamin B_1 content distribution
SG 107-35	**2.01** [±0.68][abcd]	
TAI 8	**2.03** [±0.59][abcd]	
H226	**2.04** [±0.27][abcd]	
BRA 685	**2.10** [±0.65] [abcd]	
KM397	**2.10** [±0.27][abcd]	
TMS 30572	**2.11** [±0.47][abcd]	
TAI 3	**2.16** [±0.35][abcd]	
BRA 132	**2.17** [±1.21][abcd]	
TMe-3	**2.18** [±0.22][abcd]	
KM140	**2.23** [±0.55][abcd]	
Kibandameno	**2.26** [±0.32][abcd]	
Ebwanateraka	**2.29** [±0.17][abcd]	
VEN 25	**2.39** [±0.49][abcd]	
cv. 60444	**2.41** [±0.69][abcd]	
TMS 30001	**2.46** [±0.31][abcde]	
COL 22	**2.54** [±0.48][abcde]	
11SA12	**2.59** [±0.66][abcde]	
PSE-XXX-1	**2.74** [±0.73][abcdef]	
Kibaha	**2.74** [±0.86][abcdef]	
CM 523-7	**2.81** [±0.20][abcdef]	Above the 75th percentile of vitamin B_1 content distribution
BRA 293	**2.82** [±0.47][abcdef]	
SC8	**2.86** [±0.39][abcdef]	
GUA 79	**2.87** [±0.39][abcdef]	
ARG 13	**2.94** [±0.30][abcdef]	
TMe-7	**3.08** [±0.17][bcdef]	
ECU 72	**3.17** [±1.01][bcdef]	
PER 183	**3.30** [±0.57][cdef]	
TMS 30555	**3.72** [±1.40][def]	
TMe-14	**4.45** [±0.42][ef]	
COL 1505	**4.72** [±0.69][f]	
Maximum fold difference		**4.68** [±0.69]

Supplementary Table S6. Repeated independent quantification of vitamin B_1 in 18 selected cassava accessions using a yeast bioassay.

Leaf **(A, C)** and storage root **(B, D)** vitamin B_1 contents in greenhouse-grown cassava accessions. Vitamin B_1 contents were expressed according to the fresh weight (FW) **(A, B)** and dry weight (DW) **(C, D)**. Accessions with a vitamin B_1 content below the 25th percentile of the distribution were considered as accessions with low vitamin B_1 content, whereas those with a vitamin content above the 75th percentile were considered as accessions with high vitamin B_1 content. Low, intermediate and high vitamin B_1 accessions selected for HPLC analysis are in blue-green, blue and vermilion, respectively. Average ± S.D. of 4 biological replicates except CM2177-2 (n=1), TST-XXX-18 (n=3) for leaves and CM2177-2 (n=1), TST XXX-18 (n=1), MEX 95 (n=3) for storage roots. Tukey's multiple comparison test ($p < 0.05$). Maximum fold difference (highest/lowest vitamin B_1 content) and S.D. calculated with Taylor series linearization method.

A LEAVES

Accession	Vitamin B_1 (ng mg FW^{-1})	
TAI 3	**1.66** [±0.29]^{a}	Below the 25th percentile
BRA 132	**1.90** [±0.17]^{a}	
PSE XXX-1	**2.34** [±0.48]^{ab}	
KBH 2006/18	**2.73** [±0.18]^{abc}	Between 25th and 75th percentile
cv. 60444	**2.87** [±0.17]^{abc}	
MEX 95	**2.87** [±0.81]^{abc}	
KU 50	**2.90** [±1.22]^{abc}	
CM 2177-2	**3.05**	
TST XXX-18	**3.09** [±0.23]^{abc}	
TMS 30572	**3.12** [±1.25]^{abc}	
COL 22	**3.35** [±0.76]^{abc}	
TMe-3	**3.37** [±0.75]^{abc}	
TMe-7	**3.57** [±0.47]^{abc}	
SC8	**4.06** [±1.28]^{bcd}	
ARG 13	**4.40** [±0.89]^{cde}	Above the 75th percentile
COL 1505	**4.43** [±0.91]^{cde}	
GUA 79	**5.68** [±1.07]^{de}	
Kibaha	**6.35** [±0.78]^{e}	
Maximum fold difference		**3.82** [±0.49]

B STORAGE ROOTS

Accession	Vitamin B_1 (ng mg FW^{-1})	
KBH 2006/18	**0.69** [±0.07]^{a}	Below the 25th percentile
Kibaha	**0.73** [±0.16]^{a}	
GUA 79	**0.89** [±0.21]^{ab}	Between 25th and 75th percentile
cv. 60444	**0.90** [±0.18]^{ab}	
TST XXX-18	**0.92**	
TMe-3	**0.95** [±0.10]^{abc}	
SC8	**0.97** [±0.26]^{abc}	
COL 22	**0.97** [±0.18]^{abc}	
BRA 132	**0.99** [±0.14]^{abc}	
CM 2177-2	**1.00**	
PSE XXX-1	**1.01** [±0.23]^{abc}	
KU 50	**1.01** [±0.14]^{abc}	
TMS 30572	**1.09** [±0.10]^{abc}	
TMe-7	**1.17** [±0.17]^{abc}	
MEX 95	**1.17** [±0.31]^{abc}	
TAI 3	**1.18** [±0.12]^{abc}	Above the 75th percentile
ARG 13	**1.37** [±0.14]^{bc}	
COL 1505	**1.41** [±0.36]^{c}	
Maximum fold difference		**2.03** [±0.52]

C LEAVES

Accession	Vitamin B_1 (ng mg DW^{-1})	
TAI 3	**3.71** [±0.56]^{a}	Below the 25th percentile
BRA 132	**5.14** [±0.54]^{ab}	
PSE XXX-1	**5.81** [±1.16]^{abc}	
KBH 2006/18	**6.75** [±0.90]^{abc}	Between 25th and 75th percentile
KU 50	**7.59** [±3.50]^{abcd}	
TST XXX-18	**7.62** [±1.37]^{abcd}	
MEX 95	**7.79** [±2.58]^{abcd}	
TMS 30572	**7.93** [±2.93]^{abcd}	
cv. 60444	**8.24** [±0.61]^{abcd}	
TMe-3	**8.32** [±2.28]^{abcd}	
CM 2177-2	**8.66**	
COL 22	**8.70** [±2.01]^{abcd}	
TMe-7	**9.13** [±1.61]^{abcd}	
SC8	**9.91** [±3.07]^{bcd}	
ARG 13	**11.38** [±2.00]^{cde}	Above the 75th percentile
COL 1505	**13.05** [±3.25]^{de}	
Kibaha	**17.26** [±2.48]^{ef}	
GUA 79	**19.18** [±3.23]^{f}	
Maximum fold difference		**5.17** [±0.89]

D STORAGE ROOTS

Accession	Vitamin B_1 (ng mg DW^{-1})	
TST XXX-18	**1.03**	Below the 25th percentile
Kibaha	**2.00** [±0.48]^{a}	
GUA 79	**2.10** [±0.60]^{a}	
MEX 95	**2.24** [±0.38]^{a}	
KBH 2006/18	**2.49** [±0.27]^{a}	Between 25th and 75th percentile
TMe-3	**2.55** [±0.22]^{a}	
SC8	**2.79** [±1.04]^{ab}	
COL 22	**2.82** [±0.59]^{ab}	
BRA 132	**2.90** [±0.76]^{ab}	
KU 50	**2.95** [±0.48]^{ab}	
PSE XXX-1	**3.02** [±1.53]^{ab}	
cv. 60444	**3.03** [±0.62]^{ab}	
TMS 30572	**3.05** [±0.54]^{ab}	
TMe-7	**3.10** [±0.38]^{ab}	
CM 2177-2	**3.28**	
TAI 3	**3.39** [±0.33]^{ab}	Above the 75th percentile
COL 1505	**3.73** [±0.88]^{ab}	
ARG 13	**4.31** [±0.51]^{b}	
Maximum fold difference		**2.15** [±0.30]

Supplementary Table S7. Thermal processing of cassava commercial storage roots.
Cassava commercial storage roots processed for consumption. The gain of weight during processing is expressed as a percentage of the raw material weight. Moisture content in root pieces is expressed as a percentage of the fresh weight and corresponds to the contribution of water to the fresh weight. Vitamin B_1 content was quantified using a yeast bioassay.

	RAW		BOILING			SOAKING + BOILING		
	**Moisture content in root pieces (%)**	**Vitamin B_1 content (ng mg FW^{-1})**	**Gain of weight during processing (%)**	**Moisture content in root pieces (%)**	**Vitamin B_1 content (ng mg FW^{-1})**	**Gain of weight during processing (%)**	**Moisture content in root pieces (%)**	**Vitamin B_1 content (ng mg FW^{-1})**
Root 1	59,4	1,6	+5,1	60,5	1,5	+4.2	63,9	0,5
Root 2	61,3	1,8	+1,8	60,9	1,5	+2.3	60,4	1,1
Root 3	60,6	1,6	+13,9	64,4	0,8	+3.3	68,9	0,7
Root 4	63,7	1,8	+14,8	60,2	1,2	+1.4	62,1	1,4
Average [±S.D.]	61.2 [±1.8]	1.7 [±0.1]	+8.9 [±6.4]	61.5 [±2.0]	1.3 [±0.3]	+2.8 [±1.2]	63.9 [±3.7]	0.9 [±0.4]
